# Morphological and phylogenetic analyses of *Bipolaris* species associated with Poales and Asparagales host plants in Iran

**DOI:** 10.3389/fcimb.2025.1520125

**Published:** 2025-03-18

**Authors:** Abdollah Ahmadpour, Zeinab Heidarian, Youbert Ghosta, Zahra Alavi, Fatemeh Alavi, Dimuthu S. Manamgoda, Jaturong Kumla, Samantha C. Karunarathna, Pabulo Henrique Rampelotto, Nakarin Suwannarach

**Affiliations:** ^1^ Higher Education Center of Shahid Bakeri, Urmia University, Miyandoab, Iran; ^2^ Department of Plant Protection, Faculty of Agriculture, Urmia University, Urmia, Iran; ^3^ Department of Botany, Faculty of Applied Sciences, University of Sri Jayewardenepura, Gangodawila, Sri Lanka; ^4^ Office of Research Administration, Chiang Mai University, Chiang Mai, Thailand; ^5^ Center of Excellence in Microbial Diversity and Sustainable Utilization, Chiang Mai University, Chiang Mai, Thailand; ^6^ Department of Biology, Faculty of Science, Chiang Mai University, Chiang Mai, Thailand; ^7^ Center for Yunnan Plateau Biology Resources Protection and Utilization, College of Biology and Food Engineering, Qujing Normal University, Qujing, Yunnan, China; ^8^ Bioinformatics and Biostatistics Core Facility, Institute of Basic Health Sciences, Federal University of Rio Grande do Sul, Porto Alegre, Brazil

**Keywords:** helminthosporioid fungi, morphology, seven novel species, phylogeny, Pleosporaceae, taxonomy

## Abstract

*Bipolaris* species exhibit various ecological roles, including plant pathogens, epiphytes, saprophytes, or endophytes, primarily associated with poaceous hosts, including cultivated cereals. Iran is known for its diverse climates and rich flora, which serve as a hotspot for fungal diversity. In this study, to determine the species diversity of *Bipolaris* associated with members of the Poales and Asparagales plant orders, samples with leaf and stem lesion symptoms were collected from these plants across various locations in Iran between 2010 and 2022. Based on the morphological characteristics and multi-locus phylogeny (ITS−rDNA, *GAPDH*, and *TEF1*), nine *Bipolaris* species were identified: *Bipolaris avrinica* sp. nov., *Bipolaris azarbaijanica* sp. nov., *Bipolaris banihashemii* sp. nov., *Bipolaris hedjaroudei* sp. nov., *Bipolaris hemerocallidis* sp. nov., *Bipolaris iranica* sp. nov., *Bipolaris persica* sp. nov., *Bipolaris crotonis*, and *Bipolaris salkadehensis*. *B. crotonis* represents a new record for Iran’s funga, while *B. salkadehensis* has been documented on several new hosts globally. The study provides detailed morphological descriptions and illustrations of all identified species, along with insights into their habitats, distributions, and phylogenetic relationships within the *Bipolaris* genus. This study also emphasizes the need for further research into fungal biodiversity in Iran and provides significant data on the distribution and host range of *Bipolaris* species.

## 1 Introduction

The genus *Bipolaris* was established by Shoemaker (1959) with *Bipolaris maydis* as the type species and belongs to the family Pleosporaceae (Pleosporales, Dothideomycetes, Ascomycota) (Manamgoda et al., 2011, 2012, 2014; Raza et al., 2019; Bhunjun et al., 2020). *Bipolaris* is a dematiaceous hyphomycetous genus, characterized by the production of sympodial conidiophores, straight or curved, and distoseptate conidia with the germination of end cells (Sivanesan, 1987; Manamgoda et al., 2011, 2012, 2014). Although the sexual state name *Cochliobolus* predates *Bipolaris*, a proposal to conserve the latter name was made (Rossman et al., 2013).

*Bipolaris* species exhibit diverse ecological roles as plant pathogens, epiphytes, saprophytes, or endophytes, often associated with grasses and cultivated cereals. These fungi are globally distributed and are significant plant pathogens causing diseases, like leaf spots, foliar blights, and root/foot rots, in various crops (Ellis, 1971; Sivanesan, 1987; Zhang and Li, 2009; Manamgoda et al., 2011, 2012, 2014; Tan et al., 2016; Raza et al., 2019; Bhunjun et al., 2020; Jayawardena et al., 2021; Ferdinandez et al., 2022; Khan et al., 2023; Farr et al., 2024). Certain *Bipolaris* species cause economically important plant diseases in cereal crops, such as rice brown spot (*Bipolaris oryzae*), barley and wheat common root rot or spot blotch (*Bipolaris sorokiniana*), and southern corn leaf blight diseases (*B. maydis*) (Manamgoda et al., 2014; Bhunjun et al., 2020; Jayawardena et al., 2021). In addition to grasses and cereals, *Bipolaris* species have been reported on over 60 other genera from various plant families, including Anacardiaceae, Araceae, Euphorbiaceae, Fabaceae, Malvaceae, Rutaceae, and Zingiberaceae, either as saprobes or pathogens (Ellis, 1971; Sivanesan, 1987; Manamgoda et al., 2011, 2012, 2014; Jayawardena et al., 2021; Farr et al., 2024). Furthermore, *Bipolaris cynodontis*, *B. oryzae*, and *Bipolaris setariae* have been identified as causative agents of human infections, such as lung and skin infections, allergic sinusitis, onychomycosis, keratitis, and central nervous system infections, particularly in immunocompromised individuals (da Cunha et al., 2012; Wang et al., 2016; Sharma and Nonzom, 2021). The ecological adaptability of *Bipolaris* is notable, as it thrives across a broad range of hosts, including grasses, cereals, and different dicotyledonous plants. This adaptability highlights the genus’ capability to colonize diverse environments and exploit varying ecological conditions.

The taxonomy of the *Bipolaris* genus has historically presented challenges due to its morphological variability and overlapping characteristics with other genera within the family Pleosporaceae. Early classifications were primarily based on morphological traits such as conidial shape, septum ontogeny, germination patterns, hilum morphology, and sexual morph characteristics (Ellis, 1971; Sivanesan, 1987; Alcorn, 1988; Manamgoda et al., 2011, 2012, 2014; Amaradasa et al., 2014; Tan et al., 2014; Hernández-Restrepo et al., 2018). However, the advent of molecular phylogenetics has revolutionized our understanding of *Bipolaris* taxonomy uncovering cryptic species complexes and providing new insights into the evolutionary relationships within the genus. Historically, the genera *Bipolaris*, *Curvularia*, *Exserohilum*, *Johnalcornia*, *Porocercospora*, and *Pyrenophora* were classified under the helminthosporioid fungi or graminicolous *Helminthosporium* (Sivanesan, 1987; Alcorn, 1988; Manamgoda et al., 2011, 2012, 2014, 2015; Amaradasa et al., 2014; Tan et al., 2014; Hernández-Restrepo et al., 2018; Marin-Felix et al., 2020). Recent advancements in molecular biology and phylogenetics have led to substantial taxonomic revisions within this group resulting in the recognition of new genera in the family Pleosporaceae (Amaradasa et al., 2014; Manamgoda et al., 2014; Tan et al., 2014). The genus *Bipolaris* is morphologically similar to *Curvularia* and shares the same sexual morph, *Cochliobolus*, which makes their differentiation challenging (Manamgoda et al., 2011, 2014, 2015; Marin-Felix et al., 2017a, b, 2020). However, *Bipolaris* conidia are generally longer and maintain a uniform curvature along their length, unlike the conidia of *Curvularia*. Additionally, the *Bipolaris* species lack stromata structures, as documented in several studies (Manamgoda et al., 2014, 2015; Marin-Felix et al., 2017a, b, 2020). For these reasons, integrating morphological observations with molecular methods is crucial for accurately delineating helminthosporioid fungi, identifying species, and recognizing cryptic species within *Bipolaris* (Manamgoda et al., 2014, 2015; Tan et al., 2014, 2016; Marin-Felix et al., 2017a, b, 2020; Raza et al., 2019; Ferdinandez et al., 2022). At present, the Index Fungorum (http://www.indexfungorum.org, accessed on 20 October 2024) lists 146 names under the genus *Bipolaris*, of which approximately 70 species have been reported from the orders Poales and Asparagales, as well as from other monocotyledonous plants (Manamgoda et al., 2014; Farr et al., 2024).

Iran, with its diverse climatic zones and rich flora, represents a hotspot for fungal biodiversity. Despite this ecological importance, the fungal diversity in Iran remains relatively underexplored. In recent years, efforts to study fungal communities in Iran have accelerated driven by advancements in molecular biology and an increasing recognition of Iran's critical role in global biodiversity conservation. To date, 11 species of *Bipolaris* have been recorded in Iran (Ahmadpour et al., 2011, 2012a, 2012b, 2013, 2014, 2018; Ershad, 2022). However, many of these species were identified based solely on morphological traits raising questions about their accuracy in light of recent molecular taxonomic revisions of *Bipolaris* species from other regions. This study aims to identify *Bipolaris* species associated with Poales and Asparagales hosts in Iran by integrating morphological characteristics, ecological observations, and molecular data including ITS−rDNA, *GAPDH*, and *TEF1* sequences.

## 2 Materials and methods

### 2.1 Sample collection and fungal isolation

A total of 130 samples exhibiting leaf and stem lesions were collected from various host plants in the orders Poales and Asparagales across different locations in Iran (Isfahan, Mazandaran, and West Azarbaijan Provinces) between 2010 and 2022, and the important collection information was recorded (Rathnayaka et al., 2024). Subsequently, they were brought to the laboratory for further analysis. Small sections, approximately 0.5 × 0.5 cm^2^, were cut from the interface between healthy and diseased tissue. These sections were disinfected by submerging them in a diluted bleach solution (2% sodium hypochlorite) for 2 min, followed by three thorough rinses in sterile distilled water, and then blotted dry on sterile filter paper. The disinfected sections were then transferred to Potato Dextrose Agar (PDA, 39 g/L, Merck, Germany) plates supplemented with streptomycin sulfate and penicillin G (150 ppm each). The plates were incubated at 23 ± 2°C under cool white fluorescent light with a 12-h photoperiod for 5 days. Fungi growing out from the margins of plant sections were transferred into new PDA plates and purified via single-spore or hyphal tip methods. Furthermore, infected plant samples were incubated in moist chambers at 25°C until formation of conidial mass was observed. The incubated samples were inspected under a stereomicroscope, and single spores were then transferred to PDA at 23°C–25°C using a fine sterile needle. All identified isolates were deposited as pure cultures in the fungal culture collections at the Iranian Research Institute of Plant Protection (IRAN) and Urmia University (FCCUU).

### 2.2 Morphological characterization

Mycelial disks (5 mm in diameter) were excised from the actively growing margins of 7-day-old cultures and placed on fresh PDA, Corn Meal Agar (CMA, 17 g/L, Quelab, Montreal, Canada), and Malt Extract Agar (MEA, 50 g/L, Quelab, Montreal, Canada) media plates. The plates were incubated in the dark at 25°C for 7 days. Subsequently, the characteristics of the colonies, including color, pattern, and diameter, were observed and recorded. The color of the colonies was recorded using Rayner’s (1970) color charts. The micro-morphological characteristics were observed using 10- to 14-day-old cultures on tap water agar plates with autoclaved wheat straw (TWA–wheat straw) or leaves of the host plant. The cultures were subjected to near-ultraviolet light on a 12-h diurnal cycle at 23°C–25°C, as described by Sivanesan (1987) and Hernández-Restrepo et al. (2018). Fungal structures, such as hyphae, conidiophores, conidiogenous cells, conidia, ascocarps, asci, and ascospores, were measured (20–50 measurements per structure) and photographed using an Olympus AX70 microscope with differential interference contrast (DIC) illumination from slide mounts prepared with either clear lactic acid or lactophenol cotton blue staining solutions. Images were edited with Adobe Photoshop 2020 v. 2.10.8 software (Adobe Inc., San Jose, California). Taxonomic novelties were registered in MycoBank (www.MycoBank.org; Crous et al., 2004).

### 2.3 DNA extraction, PCR amplification, and sequencing

Total genomic DNA was extracted from the mycelial mass of each isolate harvested from 10-day-old PDA Petri dishes using the method described by Ahmadpour et al. (2021). The internal transcribed spacer (ITS−rDNA) region, parts of glyceraldehyde-3-phosphate dehydrogenase (*GAPDH*), and the translation elongation factor-1 alpha (*TEF1*) genes were amplified using the primer pairs ITS1/ITS4 (White et al., 1990), gpd1/gpd2 (Berbee et al., 1999), and TEF1-983F/TEF1-2218R (Rehner and Buckley, 2005), respectively. Polymerase chain reaction (PCR) was performed in the SimpliAmp™ Thermal Cycler (Applied Biosystems™, Thermo Fisher Scientific Corp., USA) with a final volume of 30 μl. The PCR mixture comprised of 0.4 μM of each primer, 10 μl of a ready master mix (Taq DNA Polymerase 2× Master Mix Red, 2 mM MgCl_2_, Ampliqon Company, Denmark), and approximately 10 ng of DNA. The PCR amplification conditions were as follows: an initial denaturation at 95°C for 5 min, followed by 35 cycles of denaturation at 95°C for 45 s, annealing at 62°C–57°C (annealing temperature decreased by 0.5°C per cycle in the first 12 cycles) for 45 s, extension at 72°C for 45 s, and a final extension step at 72°C for 7 min. Amplicons were visualized on a 1% agarose gel stained with FluoroVue^TM^ Nucleic Acid Gel Stain (SMOBIO Technology Inc., China), and the sizes of amplicons were determined using a FluoroBand^TM^ 100 bp+3K Fluorescent DNA Ladder (SMOBIO Technology Inc., China). The amplified products were cleaned and sequenced by Macrogen Corp. (Seoul, South Korea) using the same primer sets that were used for PCR amplification. The sequences derived from this study were submitted to GenBank (**Table 1**).

**Table 1:** GenBank and culture collection accession numbers of *Bipolaris* isolates used in this study.

Species	Isolate/culture collection^a,b^	Host/Substratum	Country	GenBank accessions			References
				ITS	*GAPDH*	*TEF1*	
*Bipolaris adikaramae*	USJCC–0008^T^	*Panicum maximum*	Sri Lanka	MN535176	MT497479	MT548605	Ferdinandez et al., 2022
*B. adikaramae*	USJCC–0017	*Panicum maximum*	Sri Lanka	MT509431	MT497473	MT548601	Ferdinandez et al., 2022
*B. austrostipae*	BRIP 12490^T^	*Austrostipa verticillata*	Australia	KX452442	KX452408	KX452459	Tan et al., 2016
***B. avrinica***	**IRAN 4806C^T^**	***Setaria* sp.**	**Iran**	**PP799772**	**PP806864**	**PP806836**	**This study**
***B. avrinica***	**FCCUU 1012**	***Setaria* sp.**	**Iran**	**PP799773**	**PP806865**	**PP806837**	**This study**
*B. axonopicola*	BRIP 11740^T^	*Axonopus fissifolius*	Australia	KX452443	KX452409	KX452460	Tan et al., 2016
***B. azarbaijanica***	**IRAN 4776C^T^**	***Setaria* sp.**	**Iran**	**PP799774**	**PP806866**	**PP806838**	**This study**
***B. azarbaijanica***	**FCCUU 1010**	***Setaria* sp.**	**Iran**	**PP799775**	**PP806867**	**PP806839**	**This study**
*B. bamagaensis*	BRIP 13577^T^	*Brachiaria subquadripara*	Australia	KX452445	KX452411	KX452462	Tan et al., 2016
*B. bamagaensis*	BRIP 10711	*Dactyloctenium aegyptium*	Australia	KX452444	KX452410	KX452461	Tan et al., 2016
***B. banihashemii***	**IRAN 3389C^T^**	***Setaria* sp.**	**Iran**	**PP799777**	**PP806869**	**PP806840**	**This study**
***B. banihashemii***	**IRAN 3388C**	***Setaria* sp.**	**Iran**	**PP799778**	**PP806870**	**PP806841**	**This study**
***B. banihashemii***	**IRAN 3387C**	***Setaria* sp.**	**Iran**	**PP799779**	**PP806871**	**PP806842**	**This study**
*B. bicolor*	CBS 690.96	Unknown	Unknown	KJ909762	KM042893	KM093776	Manamgoda et al., 2014
*B. brachiariae*	CPC 28819^T^	*Brachiaria mutica*	Thailand	MF490806	MF490828	MF490850	Marin-Felix et al., 2017b
*B. chloridis*	BRIP 10965^T^	*Chloris gayana*	Australia	KJ415523	KJ415423	KJ415472	Tan et al., 2014
*B. chusqueae*	SGO 166370^T^	*Chusquea cumingii*	Chile	OM914401	OM912808	**−**	Lebeuf et al., 2023
*B. clavata*	BRIP 12530^T^	*Dactyloctenium radulans*	Australia	KJ415524	KJ415422	KJ415471	Tan et al., 2014
*B. coffeana*	BRIP 14845^IsoT^	*Coffea arabica*	Kenya	KJ415525	KJ415421	KJ415470	Tan et al., 2014
*B. cookei*	MAFF 51191	*Sorghum bicolor*	Japan	KJ922392	KM034834	KM093778	Manamgoda et al., 2014
*B. cookei*	AR5185	*Sorghum* sp.	Japan	KJ922391	KM034833	KM093777	Manamgoda et al., 2014
*B. crotonis*	CBS 274.91^IsoT^	*Eleusine indica*	Australia	KJ909768	KM034820	KM093758	Manamgoda et al., 2014
***B. crotonis***	**IRAN 4807C**	***Eleusine indica***	**Iran**	**PP799776**	**PP806868**	**−**	**This study**
*B. cynodontis*	CBS 109894^ET^	*Cynodon dactylon*	Hungary	KJ909767	KM034838	KM093782	Manamgoda et al., 2014
*B. distoseptata*	CGMCC 3.19361^T^	*Saccharum officinarum*	China	MN215628	MN264064	MN263922	Raza et al., 2019
*B. drechsleri*	CBS 136207^T^	*Microstegium vimineum*	USA	KF500530	KF500533	KM093760	Crous et al., 2013; Manamgoda et al., 2014
*B. drechsleri*	FIP 373	Ornamental grass	USA	KF500531	KF500534	KM093759	Crous et al., 2013
*B. fujianensis*	CGMCC 3.2088^T^	*Cunninghamia lanceolata*	China	MN595057	MW051017	MT966888	Zhang et al., 2024
*B. fujianensis*	cfsb5	*Cunninghamia lanceolata*	China	MT974094	MT993889	MW051019	Zhang et al., 2024
*B. gigantea*	NRRL 66763	*Microstegium vimineum*	USA	KM507761	**−**	MN894581	Lane et al., 2020
*B. gossypina*	BRIP 14840^T^	*Gossypium* sp.	Kenya	KJ415528	KJ415418	KJ415467	Tan et al., 2014
***B. hedjaroudei***	**IRAN 4805C^T^**	***Setaria* sp.**	**Iran**	**PP799788**	**PP806880**	**PP806851**	**This study**
***B. hedjaroudei***	**FCCUU 1013**	***Setaria* sp.**	**Iran**	**PP799789**	**PP806881**	**PP806852**	**This study**
*B. heliconiae*	BRIP 17186^T^	*Heliconia psittacorum*	Australia	KJ415530	KJ415417	KJ415465	Tan et al., 2014
***B. hemerocallidis***	**IRAN 4774C^T^**	***Hemerocallis fulva***	**Iran**	**PP799780**	**PP806872**	**PP806843**	**This study**
***B. hemerocallidis***	**FCCUU 1011**	***Hemerocallis fulva***	**Iran**	**PP799781**	**PP806873**	**PP806844**	**This study**
*B. heveae*	CBS 241.92^T^	*Hevea* sp.	Nigeria	KJ909763	KM034843	KM093791	Manamgoda et al., 2014
***B. iranica***	**IRAN 4775C^T^**	***Cynodon dactylon***	**Iran**	**PP799782**	**PP806874**	**PP806845**	**This study**
***B. iranica***	**FCCUU 1005**	***Sorghum halepense***	**Iran**	**PP799783**	**PP806875**	**PP806846**	**This study**
***B. iranica***	**FCCUU 1006**	***Arundo donax***	**Iran**	**PP799784**	**PP806876**	**PP806847**	**This study**
***B. iranica***	**FCCUU 1007**	***Echinochloa colona***	**Iran**	**PP799785**	**PP806877**	**PP806848**	**This study**
***B. iranica***	**FCCUU 1008**	***Hordeum vulgare***	**Iran**	**PP799786**	**PP806878**	**PP806849**	**This study**
***B. iranica***	**FCCUU 1009**	***Triticum aestivum***	**Iran**	**PP799787**	**PP806879**	**PP806850**	**This study**
*B. louisemackiae*	BRIP 14812b^T^	Unknown	Australia	OR271904	OR269435	**−**	Tan and Shivas, 2023
*B. luttrellii*	BRIP 14643^T^	*Dactyloctenium aegyptium*	Australia	AF071350	AF081402	KJ415464	Yun et al., 1999; Tan et al., 2014
*B. marantae*	COAD 2068^T^	*Maranta leuconeura*	Brazil	KX365749	KX907136	**−**	Lourenço et al., 2017
*B. mariehareliae*	BRIP 75357a^T^	*Cycas candida*	Australia	OR271905	OR269436	OR269441	Tan and Shivas, 2023
*B. maryandersoniae*	BRIP 72520b^T^	*Leersia hexandra*	Australia	OR271906	OR269434	OR269442	Tan and Shivas, 2023
*B. maydis*	CBS 137271/C5 ^NT^	*Zea mays*	USA	AF071325	KM034846	KM093794	Berbee et al., 1999; Manamgoda et al., 2014
*B. maydis*	AR5182	*Sorghum bicolor*	Japan	KM230388	KM034844	KM093792	Manamgoda et al., 2014
*B. microconidica*	CGMCC 3.1936^T^	*Saccharum officinarum*	China	MN215630	MN264066	MN263924	Raza et al., 2019
*B. microconidica*	LC12040	*Saccharum officinarum*	China	MN215631	MN264067	MN263925	Raza et al., 2019
*B. microlaenae*	BRIP 15613^T^	*Microlaena stipoides*	Australia	JN601032	JN600974	JN601017	Manamgoda et al., 2011
*B. microstegii*	CBS 132550^T^	*Microlaena vimineum*	USA	JX089579	JX089575	KM093756	Manamgoda et al., 2014
*B. microstegii*	AR5192	*Microlaena vimineum*	USA	KM230391	KM034819	KM093757	Manamgoda et al., 2014
*B. omanensis*	SQUCC 13928^T^	*Hibiscus* sp.	Oman	MK072962	MK089803	**−**	Al Dughaishi et al., 2018
*B. oryzae*	MFLUCC 10-0715 ^NT^	*Oryza sativa*	Thailand	JX256416	JX276430	JX266585	Manamgoda et al., 2012
*B. oryzae*	MAFF 235499	*Oryza sativa*	Japan	KJ922383	KM042897	KM093789	Manamgoda et al., 2014
*B. panici-miliacei*	CBS 199.29 ^LT^	*Panicum miliaceum*	Japan	KJ909773	KM042896	KM093788	Manamgoda et al., 2014
*B. peregianensis*	BRIP 12790^T^	*Cynodon dactylon*	Australia	JN601034	JN600977	JN601022	Manamgoda et al., 2011
*B. peregianensis*	DAOM 221998	*Cynodon dactylon*	Australia	KJ922393	KM034849	KM093797	Manamgoda et al., 2011
***B. persica***	**IRAN 4777C^T^**	***Cynodon dactylon***	**Iran**	**PP799790**	**PP806882**	**PP806853**	**This study**
***B. persica***	**FCCUU 1004**	***Cynodon dactylon***	**Iran**	**PP799791**	**PP806883**	**PP806854**	**This study**
*B. petchii*	USJCC–0007^T^	*Ischaemum* sp.	Sri Lanka	MN535174	MT497476	MT548603	Ferdinandez et al., 2022
*B. petchii*	USJCC–0018	*Ischaemum* sp.	Sri Lanka	MT509432	MT497475	MT548602	Ferdinandez et al., 2022
*B. pluriseptata*	BRIP 14839^IsoT^	*Eleusine coracana*	Zambia	KJ415532	KJ415414	KJ415461	Tan et al., 2014
*B. sacchari*	ICMP 6227	*Oplismenus imbecillis*	New Zealand	KJ922386	KM034842	KM093785	Manamgoda et al., 2014
*B. saccharicola*	CBS 155.26^T^	*Saccharum officinarum*	Unknown	KY905674	KY905686	KY905694	Marin-Felix et al., 2017a
*B. saccharicola*	CBS 324.64	*Saccharum officinarum*	Unknown	HE792932	KY905692	KY905699	Marin-Felix et al., 2017a
***B. salkadehensis***	**IRAN 3382C**	***Scirpus acutus***	**Iran**	**PP799794**	**PP806886**	**PP806857**	**This study**
***B. salkadehensis***	**IRAN 3383C**	***Sorghum halepense***	**Iran**	**PP799795**	**PP806887**	**PP806858**	**This study**
***B. salkadehensis***	**FCCUU 1001**	***Arundo donax***	**Iran**	**PP799796**	**PP806888**	**PP806859**	**This study**
***B. salkadehensis***	**FCCUU 1002**	***Setaria* sp.**	**Iran**	**PP799797**	**PP806889**	**PP806860**	**This study**
***B. salkadehensis***	**FCCUU 1003**	***Hordeum vulgare***	**Iran**	**PP799798**	**PP806890**	**PP806861**	**This study**
***B. salkadehensis***	**Bi 1= IRAN 3385C^T^**	***Sparganium erectum***	**Iran**	AB675490	**PP806891**	**PP806862**	Ahmadpour et al., 2012a, **This study**
***B. salkadehensis***	**Bi 4 = IRAN 3386C**	***Cladium mariscus***	**Iran**	AB675491	**PP806892**	**PP806863**	Ahmadpour et al., 2012a; **This study**
*B. salviniae*	IMI 228224^ET^	*Salvinia auriculata*	Brazil	KJ922390	KM034829	KM093772	Manamgoda et al., 2014
*B. salviniae*	BRIP 16571^LT^	*Salvinia auriculata*	Brazil	KJ415535	KJ415411	KJ415457	Tan et al., 2014
*B. secalis*	BRIP 14453^IsoLT^	*Secale cereale*	Argentina	KJ415537	KJ415409	KJ415455	Tan et al., 2014
*B. setariae*	CPC 28802	*Imperata cylindrica*	Thailand	MF490811	MF490833	**−**	Marin-Felix et al., 2017b
*B. setariae*	CBS 141.31	Unknown	Unknown	EF452444	EF513206	**−**	Andrie et al., 2008
*B. setariae*	LC12047	*Saccharum officinarum*	China	MN215632	MN264068	MN263926	Raza et al., 2019
*B. shoemakeri*	BRIP 15929^T^	*Ischaemum rugosum* var*. segetum*	Australia	KX452453	KX452419	KX452470	Tan et al., 2016
*B. shoemakeri*	BRIP 15806	*Ischaemum rugosum* var. *segetum*	Australia	KX452452	KX452418	KX452469	Tan et al., 2016
*B. simmondsii*	BRIP 12030^T^	*Zoysia macrantha*	Australia	KX452454	KX452420	KX452471	Tan et al., 2016
*B. sivanesaniana*	BRIP 15847^T^	*Paspalidium distans*	Australia	KX452455	KX452421	KX452472	Tan et al., 2016
*B. sivanesaniana*	BRIP 15822	*Setaria sphaecelata*	Australia	KX452456	KX452422	KX452473	Tan et al., 2016
*B. sorokiniana*	CBS 480.74	*Tribulus terrestris*	South Africa	KJ909771	KM034827	KM093768	Manamgoda et al., 2014
*B. sorokiniana*	CBS 110.14	*Hordeum* sp.	USA	KJ922381	KM034822	KM093763	Manamgoda et al., 2014
*B. stenospila*	CBS 156.36	Unknown	Unknown	MH855749	**−**	**−**	Vu et al., 2019
*B. subramanianii*	BRIP 16226^T^	*Setaria sphacelata*	Australia	KX452457	KX452423	KX452474	Tan et al., 2016
*B. urochloae*	ATCC 58317	*Urochloa panicoides*	Australia	KJ922389	KM230396	KM093770	Manamgoda et al., 2014
*B. variabilis*	CBS 127716^T^	*Pennisetum clandestinum*	Argentina	KY905676	KY905688	KY905696	Marin-Felix et al., 2017a
*B. variabilis*	CBS 127736	*Pennisetum clandestinum*	Brazil	KY905677	KY905689	-	Marin-Felix et al., 2017a
*B. victoriae*	CBS 327.64^T^	*Avena sativa*	USA	KJ909778	KM034811	KM093748	Manamgoda et al., 2014
*B. victoriae*	DAOM 147449	*Avena sativa*	USA	KJ909785	KM034812	KM093749	Manamgoda et al., 2014
*B. woodii*	BRIP 12239^T^	*Paspalidium caespitosum*	Australia	KX452458	KX452424	KX452475	Tan et al., 2016
*B. yamadae*	CBS 202.29^ET^	*Panicum miliaceum*	Japan	KJ909779	KM034830	KM093773	Manamgoda et al., 2014
*B. zeae*	BRIP 11512^IsoPT^	*Zea mays*	USA	KJ415538	KJ415408	KJ415454	Tan et al., 2014
*B. zeae*	DAOM 211267	*Triticum* sp.	Canada	KJ909787	KM034818	KM093755	Manamgoda et al., 2014
*B. zeicola*	FIP 532^ET^	*Zea mays*	USA	KM230398	KM034815	KM093752	Manamgoda et al., 2014
*B. zeicola*	AR5166	*Sorghum* sp.	USA	KJ909788	KM034813	KM093750	Manamgoda et al., 2014
*Curvularia affinis*	CBS 154.34^T^	unknown	Indonesia	KJ909780	KM230401	KM196566	Manamgoda et al., 2015
*C. lunata*	CBS 730.96^T^	Human lung biopsy	USA	JX256429	JX276441	JX266596	Manamgoda et al., 2012

Newly generated sequences are in bold.

a Culture collections: ATCC American Type Culture Collection, Virginia, USA; BRIP Queensland Plant Pathology Herbarium, Queensland, Australia; CBS, CBS-KNAW Fungal Biodiversity Centre, Utrecht, The Netherlands; CGMCC China General Microbiological Culture Collection, Institute of Microbiology, Chinese Academy of Sciences, Beijing, China; COAD Coleção Octávio de Almeida Drumond housed at Universidade Federal de Viçosa; CPC Working collection of P.W. Crous, housed at the Westerdijk Fungal Biodiversity Institute, Utrecht, The Netherlands; AR ans FIP Isolates housed in Mycology and Nematology Genetic Diversity and Biology Laboratory, United States Department of Agriculture, Agricultural Research Service, Beltsville, Maryland; FCCUU the fungal culture collections of Urmia University, Iran; ICMP International Collection of Micro-organisms from Plants, Landcare Research, Auckland, New Zealand; IMI International Mycological Institute, Kew, UK; IRAN Iranian Fungal Culture Collection, Iranian Research Institute of Plant Protection, Iran; LC: Personal culture collection of Prof. Lei Cai housed in State Key Laboratory of Mycology, Institute of Microbiology, Beijing, China; MAFF Ministry of Agriculture, Forestry and Fisheries, Tsukuba, Ibaraki, Japan; MFLUCC Mae Fah Luang University Culture Collection, Thailand; NRRL USDA Agricultural Research Service Culture Collection, USA; SQUCC Sultan Qaboos University Culture Collection, Muscat, Oman; USJCC University of Sri Jayewardenepura Culture Collection, Sri Lanka. ^b T, ET, IsoT, IsoLT, IsoPT, LT^ and ^NT^ indicate ex-type, ex-epitype, ex-isotype, ex-isolectotype, ex-isoparatype, ex-lectotype and ex-neotype strains, respectively.

### 2.4 Sequence alignments and phylogenetic analyses

The initial identification of the isolates involved utilizing newly generated sequences of ITS−rDNA, *GAPDH*, and *TEF1* with the NCBI Basic Local Alignment Search Tool (BLAST) (www.ncbi.nlm.nih.gov/blast/). Subsequently, pairwise sequence comparisons were performed between novel species and their closely related taxa using the same BLAST tool. DNA sequences from the type or representative species were obtained from GenBank (**Table 1**) and used in the analyses. A multi-locus phylogenetic analysis was conducted on a combined dataset comprising the three genes/regions (ITS−rDNA + *GAPDH* + *TEF1*). Multiple sequence alignment was done using the online alignment tool MAFFT version 7 (https://mafft.cbrc.jp/alignment/server/) (Katoh et al., 2019). The best-fit substitution models were determined with the Akaike Information Criterion (AIC) in MrModeltest 2.3 (Nylander, 2004). The maximum likelihood (ML) and maximum parsimony (MP) analyses were conducted via the CIPRES Science Gateway portal (accessible at https://www.phylo.org/) (Miller et al., 2012) using RAxML-HPC BlackBox v. 8.2.12 (utilizing the GTR + GAMMA model and 1,000 bootstrapping iterations) (Stamatakis, 2014) and PAUP on ACCESS v. 4.a168 (using the heuristic search option and branch swapping with the tree–bisection–reconnection (TBR) algorithm with 1,000 bootstrapping replicates) (Swofford, 2002) tools, respectively. Descriptive tree statistics [tree length (TL), consistency index (CI), retention index (RI), and homoplasy index (HI)] were calculated for trees generated in the parsimony analysis. Bayesian phylogenetic inference (BI) and Bayesian posterior probabilities (BPP) were conducted in MrBayes v. 3.2.7 (Ronquist et al., 2012) with the Markov chain Monte Carlo (MCMC) method (four chains, 1,000,000 generations, 1,000 sampling frequency, and 25% burn-in phase). In all phylogenetic analyses, *Curvularia affinis* (CBS 154.34) and *Curvularia lunata* (CBS 730.96) were used as the outgroup taxa (Manamgoda et al., 2014; Tan et al., 2016; Bhunjun et al., 2020; Ferdinandez et al., 2022). The generated phylogenetic trees were viewed using FigTree v. 1.4.4 (Rambaut, 2019) and further edited using graphic design software, Adobe Illustrator^®^ CC 2020.

### 2.5 Genealogical Concordance Phylogenetic Species Recognition analysis

Genealogical Concordance Phylogenetic Species Recognition (GCPSR) was used to test for significant recombinant events (Quaedvlieg et al., 2014). Three-locus concatenated datasets (ITS−rDNA + *GAPDH* + *TEF1*) with closely related species were used for the analyses. The data were analyzed using SplitsTree 5 software employing the pairwise homoplasy index (PHI or Φw) test (Bruen et al., 2006; Huson and Bryant, 2006). PHI test results indicating a value less than 0.05 (Φw < 0.05) suggest the presence of significant recombination within the dataset. To visualize the relationships between novel taxa and their closely related counterparts, split graphs were constructed using concatenated datasets. The LogDet transformation and split decomposition options were used for this purpose.

## 3 Results

### 3.1 Phylogenetic analyses

**Figure 1:**
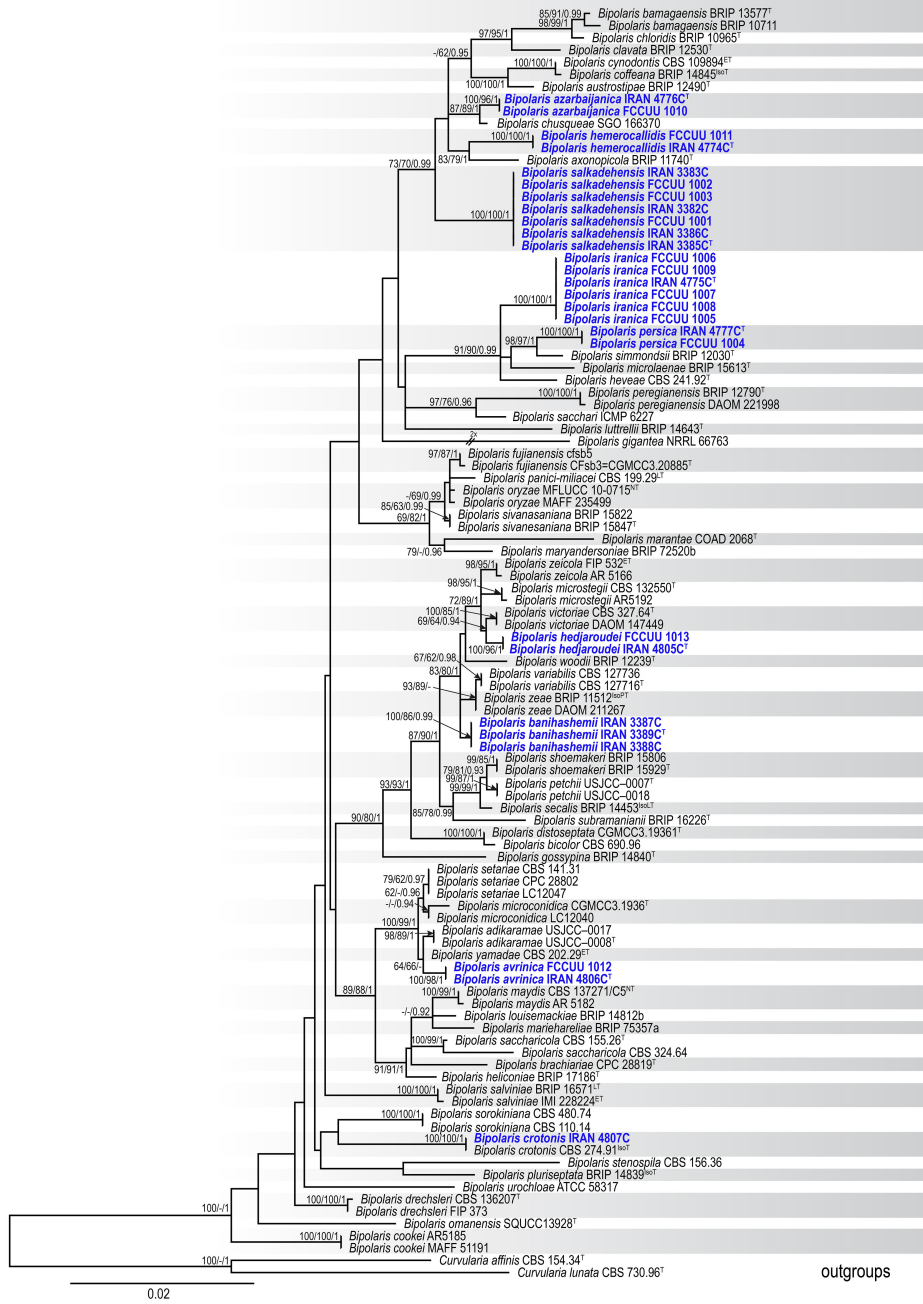
Maximum likelihood (ML) tree of *Bipolaris* species based on the dataset of ITS−rDNA, *GAPDH*, and *TEF1*. Bootstrap support values of the ML and maximum parsimony (MP) (MLBS/MPBS) values ≥60% and Bayesian posterior probabilities (BIPP) ≥0.90 are given at the nodes. The tree is rooted with *Curvularia affinis* (CBS 154.34) and *C. lunata* (CBS 730.96), and new species are indicated in blue boldface. The scale bar indicates the number of nucleotide substitutions. ^T, ET, IsoT, IsoLT, IsoPT, LT^ and ^NT^ indicate ex-type, ex-epitype, ex-isotype, ex-isolectotype, ex-isoparatype, ex-lectotype, and ex-neotype strains, respectively.

A total of 85 isolates were obtained from various hosts (Poales and Asparagales plants). All isolates were examined based on their morphology. Representative isolates were then selected from various plant hosts for phylogenetic analyses. PCR amplifications produced DNA fragments of approximately 540 bp for ITS−rDNA, 545 bp for *GAPDH*, and 850 bp for *TEF1*. A total of 104 ITS−rDNA, 102 *GAPDH*, and 95 *TEF1* sequences were subjected to multiple sequence alignment (nucleotides + gaps) resulting in 505-, 494-, and 898-character datasets, respectively. A combination of three gene sequences from 104 strains yielded a dataset with 1,897 characters, of which, 1,472 characters were constant, 112 characters were variable and parsimony uninformative, and 313 were parsimony informative. The most parsimonious tree yielded the following metrics: TL = 937, CI = 0.574, RI = 0.842, HI = 0.426. The nucleotide substitution model GTR + I + G was identified by MrModeltest 2.3 for all ITS−rDNA, *GAPDH*, and *TEF1* datasets. The ML, MP, and BI phylogenetic analyses produced trees with similar topology and showed no significant conflicts. The combined dataset analysis of RAxML generated the best-scoring tree (**Figure 1**) with a final ML optimization likelihood value of −8,235.239283. Estimated base frequencies were as follows: A = 0.229913, C = 0.302740, G = 0.236579, T = 0.230767; substitution rates AC = 1.001686, AG = 2.714677, AT = 1.255930, CG = 0.832074, CT = 6.025850, GT = 1.000000; gamma distribution shape parameter α = 0.731765. Based on morphological characteristics and multi-locus phylogeny (ITS−rDNA, *GAPDH*, and *TEF1*), nine *Bipolaris* species were identified: *Bipolaris avrinica* sp. nov., *B. azarbaijanica* sp. nov., *B. banihashemii* sp. nov., *B. hedjaroudei* sp. nov., *B. hemerocallidis* sp. nov., *B. iranica* sp. nov., *B. persica* sp. nov., *Bipolaris crotonis*, and *B. salkadehensis*. *B. crotonis* is a new record for Iran’s funga. Also, the phylogenetic relationship of *B. salkadehensis* with related species was re-defined using sequences from three genomic regions, and several new hosts were identified for this species worldwide. All identified taxa clustered with high statistical support values in the phylogenetic tree (**Figure 1**). Each species was thoroughly illustrated, described, and discussed in terms of morphology, habitat, distribution, and phylogenetic relationships with other *Bipolaris* species.

### 3.2 Taxonomy

**Figure 2:**
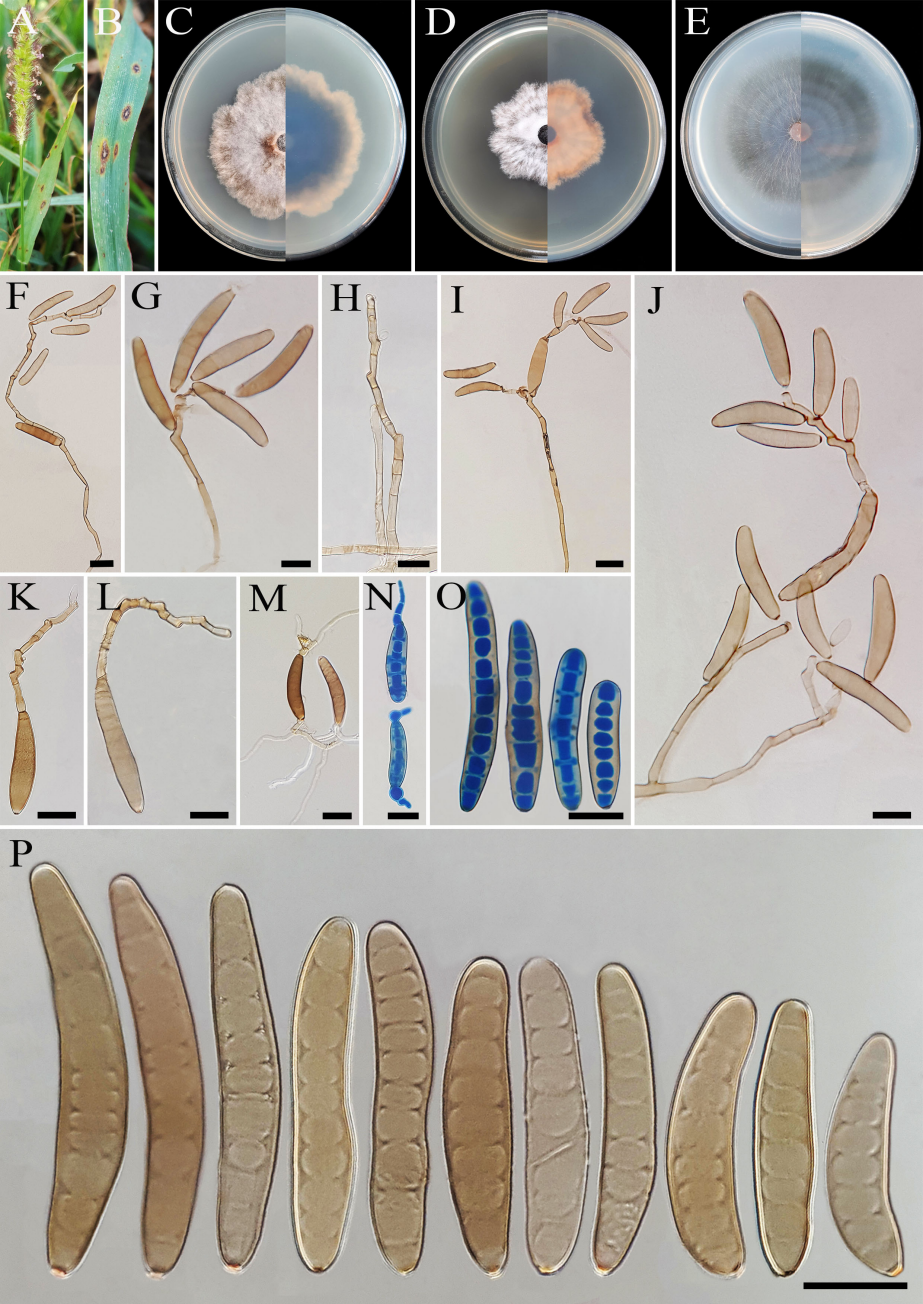
*Bipolaris avrinica* (IRAN 4806C). **(A, B)** Lesions on host leaf (*Setaria* sp.). **(C−E)** Colonies (front and reverse) on PDA **(C)**, MEA **(D)**, and CMA **(E)** media after 7 days. **(F–H)** Conidiophores. **(I–L)** Conidia with secondary sporulation. **(M, N)** Germinated conidia. **(O, P)** Conidia. Scale bars: **(F−P)** = 20 μm.

***Bipolaris avrinica*** A. Ahmadpour, Z. Heidarian, Y. Ghosta, Z. Alavi & F. Alavi, sp. nov. (**Figure 2**).

MycoBank No: MB 854730

*Etymology:* The name refers to Avrin Mountain, located in Khoy County, West Azarbaijan Province, where the holotype was collected.

*Diagnosis*: Differs from *Bipolaris adikaramae* and *B. yamadae* by the abundant production of secondary conidiophores and conidia in culture media.

*Type:* IRAN, West Azarbaijan Province, Khoy County, on infected leaves of *Setaria* sp. (Poaceae, Poales), 10 September 2020, A. Ahmadpour, (IRAN 18493F, **holotype**, dried culture; **ex-type** culture IRAN 4806C).

*Description*: Lesions on infected leaves of *Setaria* sp., 1- to 10-mm long, gray color at the center with dark brown margins. Sexual morph: Undetermined. Asexual morph: On TWA *Hyphae* 2- to 5-μm wide, pale brown to brown, smooth, septate, branched. *Conidiophores* (125–)185–500(–600) × 4–6 µm ( $\bar{x}$ ± SD = 342.5 ± 157.5 × 5 ± 1 μm, *n* = 50), mononematous, semi- to macronematous, arising singly or rarely in groups, unbranched, straight to flexuous, septate, geniculate, pale brown to brown, paler toward the apex, rarely swollen at the base. Secondary conidiophores are frequently formed in culture media and conidia attached to primary conidiophores. *Conidiogenous cells* (6–)8–21(–16) × 4–7 μm ( $\bar{x}$ ± SD = 14.5 ± 6.5 × 5.5 ± 1.5 μm, *n* = 50), mono- to polytretic, sympodial proliferation, integrated, terminal or intercalary, subcylindrical to slightly swollen, pale brown to brown, smooth-walled, with thickened and darkened scars. *Conidia* (45–)50–87(–100) × 10–13 µm ( $\bar{x}$ ± SD = 68.5 ± 18.5 × 12 ± 1 μm, *n* = 50), pale brown to brown, smooth walled, straight to curved, fusoid to cylindrical, occasionally ellipsoidal, tapering toward rounded ends, (6–)7–10(–11)-distoseptate, germinated mono- or bipolar; hila 1.5- to 2.5-μm wide, inconspicuous, flat, thickened, and darkened. *Stroma*, *chlamydospores*, and *microconidiation* were not observed.

*Culture characteristics*: Colonies on PDA reaching 50 mm in diameter after 7 days at 25°C in the dark, circular, margin irregular, cottony appearance, gray with white to gray aerial mycelia; reverse gray olivaceous. Colonies on MEA reaching 38-mm diameter, circular, margin irregular, cottony appearance, white with white aerial mycelia; reverse brown to pale brown from the center to the margin. Colonies on CMA reaching 52 mm in diameter, circular, margin entire, hairy appearance with concentric rings, gray with sparse white to gray aerial mycelia; reverse olivaceous brown at the center and a hyaline margin.

*Additional material examined*: IRAN, West Azarbaijan Province, Khoy County, on infected leaves of *Setaria* sp. (Poaceae, Poales), 10 September 2020, A. Ahmadpour, isolate FCCUU 1012.

*Host and distribution*: *Setaria* sp. in Iran (this study).

**Figure 3:**
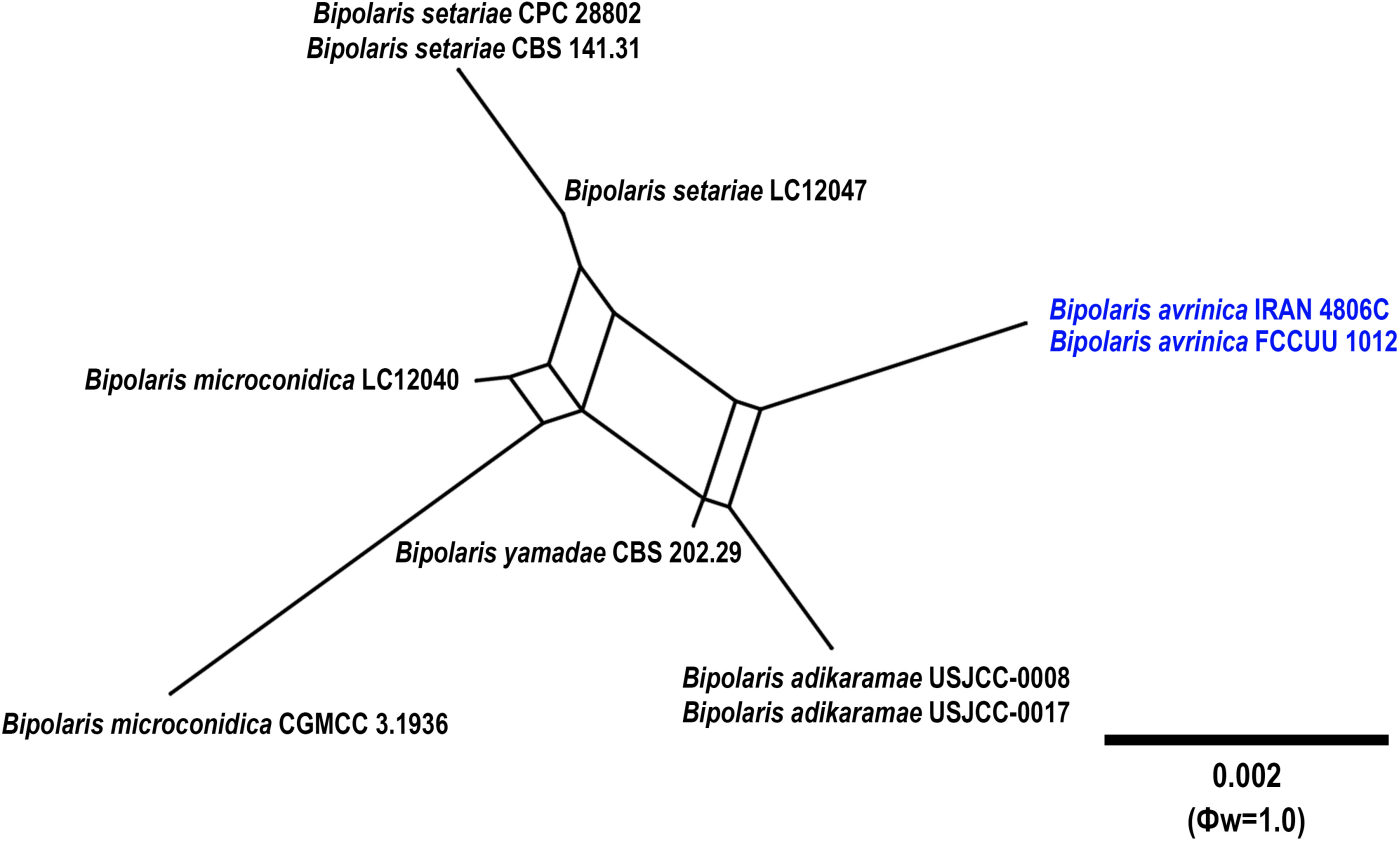
Split graphs showing the results of PHI test of *Bipolaris avrinica* with their most closely related species (Φw = 1.0). The new taxa are shown in bold blue.

*Notes:* Based on the phylogenetic analyses, *B. avrinica* is closely related to *B. adikaramae* and *B. yamadae* (MLBS/MPBS/BIPP = 100/98/1.0) (**Figure 1**). A comparison of nucleotide differences in ITS−rDNA, *GAPDH*, and *TEF1* indicates that *B. avrinica* (IRAN 4806C) differs from *B. adikaramae* (USJCC–0008) by 1/511 bp [0.19%, with one gap (0%)] in ITS−rDNA, 3/550 bp (0.54%) in *GAPDH*, and 3/763 bp (0.39%) in *TEF1* and from *B. yamadae* (CBS 202.29) by 1/511 bp [0.54%, with one gap (0%)] in ITS−rDNA, 4/480 bp (0.83%) in *GAPDH*, and 4/763 bp (0.52%) in *TEF1*. The PHI analysis confirms that *B. avrinica* shows no significant genetic recombination with closely related species (Φw = > 0.05, **Figure 3**). *Bipolaris avrinica* can be differentiated by its abundant production of secondary conidiophores and conidia in cultures, a feature absent in *B. adikaramae* and *B. yamadae*. Additionally, *B. avrinica* has smaller conidia [(45–)50–87(–100) × 10–13 μm] compared to *B. yamadae* [(60–)65–100(–120) × (12–)14–18 μm] (Manamgoda et al., 2014; Ferdinandez et al., 2022). The production of secondary conidiophores and secondary conidia has been observed in *B. cookei* and *B. microstegii* grown on culture media (Manamgoda et al., 2014). However, *B. avrinica* is phylogenetically distinct from these species (**Figure 1**). *Bipolaris yamadae* has been reported from several hosts, including *Oryza* sp., *Euphorbia* sp., *Panicum* spp. (*P. capillare*, *P. implicatum*, *P. maximum*, and *P. miliaceum*), *Saccharum officinarum*, and *Setaria plicata* (Manamgoda et al., 2014; Marin-Felix et al., 2017a; Farr et al., 2024). *Bipolaris adikaramae* has been isolated from yellow lesions on the leaf of *Panicum maximum* in Sri Lanka (Ferdinandez et al., 2022). Based on morphological and molecular evidence, we propose *B. avrinica* as a new species.

**Figure 4:**
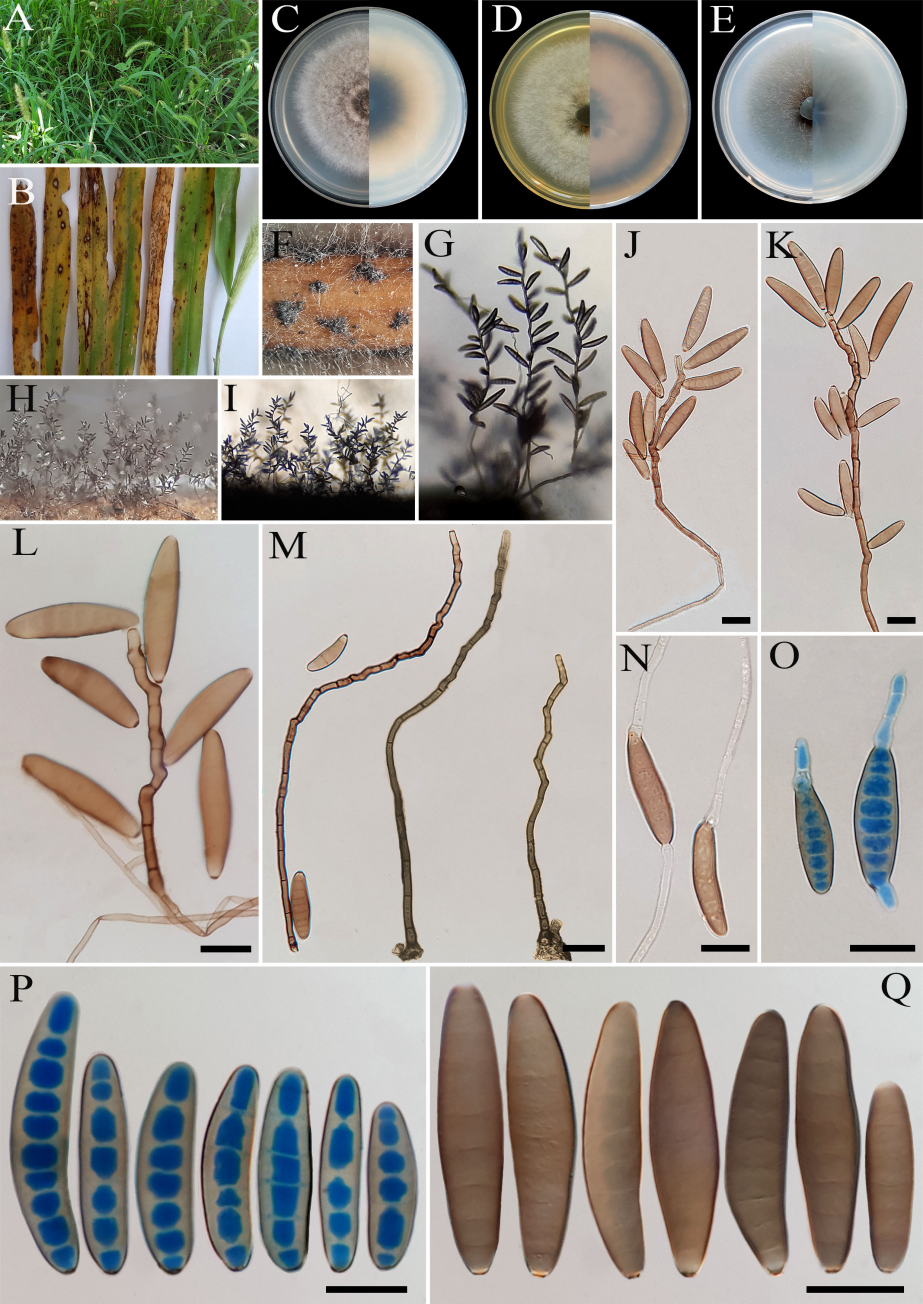
*Bipolaris azarbaijanica* (IRAN 4776C). **(A, B)** Lesions on host leaf (*Setaria* sp.). **(C−E)** Colonies (front and reverse) on PDA **(C)**, MEA **(D)**, and CMA **(E)** media after 7 days. (**F−I)** Sporulation pattern on TWA medium. **(J–M)** Conidiophores. **(N, O)** Germinated conidia. **(P, Q)** Conidia. **(J−Q)** Scale bars = 20 μm.

***Bipolaris azarbaijanica*** A. Ahmadpour, Z. Heidarian, Y. Ghosta, Z. Alavi & F. Alavi, sp. nov. (**Figure 4**).

MycoBank No: MB 854731

*Etymology*: The name refers to the West Azarbaijan Province, where the holotype was collected.

*Diagnosis*: Differs from *Bipolaris chusqueae* by the shape (fusoid to cylindrical) and size (longer and wider) of conidia.

*Type*: IRAN, West Azarbaijan Province, Salmas County, on leaves of *Setaria* sp. (Poaceae, Poales), 10 September 2015, A. Ahmadpour/Z. Heidarian, (IRAN 18208F, **holotype**, dried culture; **ex-type** culture IRAN 4776C).

*Description*: Leaf spots on *Setaria* sp., 1- to 10-mm long, gray at the center with a red-brown margin. Sexual morph: Undetermined. Asexual morph: On TWA: *Hyphae* 3- to 5-μm wide, pale brown to brown, smooth, septate, branched. *Conidiophores* (112–)140–300(–450) × 5–7 µm ( $\bar{x}$ ± SD = 220 ± 80 × 6 ± 1 μm, *n* = 50), mononematous, semi- to macronematous, arising singly or in groups, unbranched, straight to flexuous, septate, geniculate, pale brown to brown, paler toward the apex, swollen at the base. *Conidiogenous cells* (8–)10–22(–25) × 5–8 μm ( $\bar{x}$ ± SD = 16 ± 6 × 6.5 ± 1.5 μm, *n* = 50), mono- to polytretic, sympodial proliferation, integrated, terminal or intercalary, subcylindrical to slightly swollen, pale brown to brown, smooth walled to slightly verruculose, with thickened and darkened scars. *Conidia* (44–)50–80(–84) × 11–15 µm ( $\bar{x}$ ± SD = 65 ± 15 × 13 ± 2 μm, *n* = 50), pale brown to brown, smooth walled, straight to slightly curved, broadly fusoid to cylindrical, occasionally ellipsoidal to clavate, tapering toward the rounded ends, apical and basal cells paler than the median cells, (4–)5–9(–10)-distoseptate, germination mono- or bipolar; hila 2- to 3-μm wide, flat to slightly protuberant, thickened, and darkened. *Stroma*, *chlamydospores*, and *microconidiation* were not observed.

*Culture characteristics*: Colonies on PDA reaching 73 mm in diameter after 7 days at 25°C in the dark, circular, margin entire, gray at the center with white to gray aerial mycelia, white at the margin; reverse olivaceous gray at the center, margin pale brown. Colonies on MEA reaching 68 mm in diameter, circular, margin entire, cottony appearance, gray at the center, white at the margin with white aerial mycelia; reverse brown to pale brown. Colonies on CMA reaching 65 mm in diameter, circular, margin entire, hairy appearance, olivaceous gray with sparse white to gray aerial mycelia; reverse olivaceous gray at the center and a hyaline margin.

*Additional material examined*: Iran, West Azarbaijan Province, Salmas County, on leaves of *Setaria* sp. (Poaceae, Poales), 10 September 2015, A. Ahmadpour/Z. Heidarian, isolate FCCUU 1010.

*Host and distribution*: *Setaria* sp. in Iran (this study).

**Figure 5:**
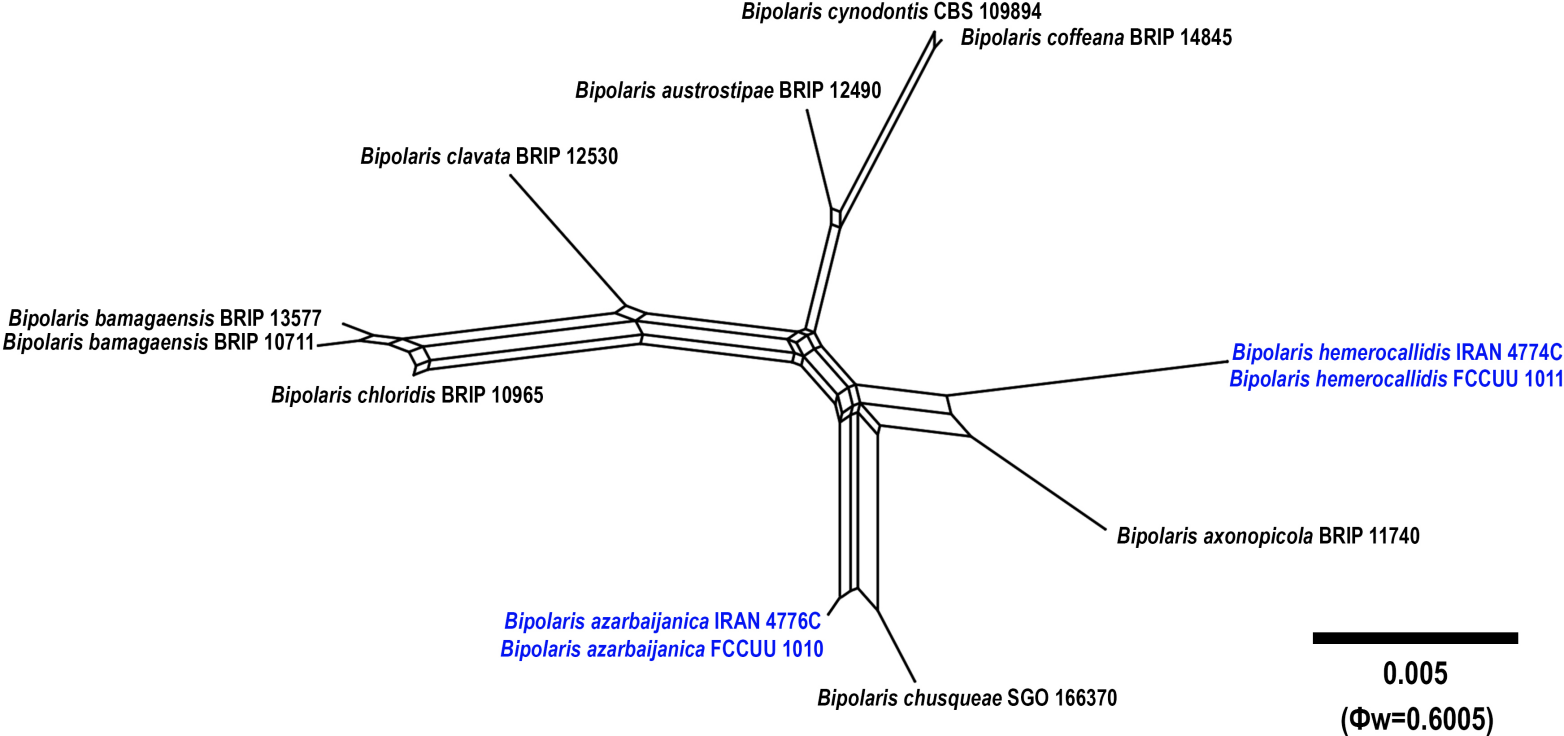
Split graphs showing the results of PHI test of *Bipolaris azarbaijanica* and *B. hemerocallidis* with their most closely related species (Φw = 0.6005). The new taxa are shown in bold blue.

*Notes: Bipolaris azarbaijanica* is phylogenetically closely related to *B. chusqueae* (MLBS/MPBS/BIPP = 100/96/1.0) (**Figure 1**). The pairwise DNA sequence comparison revealed that *B. azarbaijanica* is distinct from *B. chusqueae*. A comparison of nucleotide differences in ITS−rDNA and *GAPDH* indicates that *B. azarbaijanica* (IRAN 4776C) differs from *B. chusqueae* (SGO 166370) by 3/525 bp (0.57%) in ITS−rDNA and 6/531 bp (1.12%) in *GAPDH*. The PHI analysis confirms that *B. azarbaijanica* has no significant genetic recombination with closely related species (Φw = > 0.05, **Figure 5**). Morphologically, *B. azarbaijanica* can be differentiated by the shape of the conidia (broadly fusoid to cylindrical *vs.* subcylindrical to narrowly clavate in *B. chusqueae*), and longer and wider conidia [(44–)50–80(–84) × 11–15 µm *vs.* (17–)26–50(–68) × 10–12(–15) μm in *B. chusqueae*] (Lebeuf et al., 2023). *Bipolaris chusqueae* has been reported from *Chusquea cumingii* (Bambusoideae, Poales) in Chile (Lebeuf et al., 2023).

**Figure 6:**
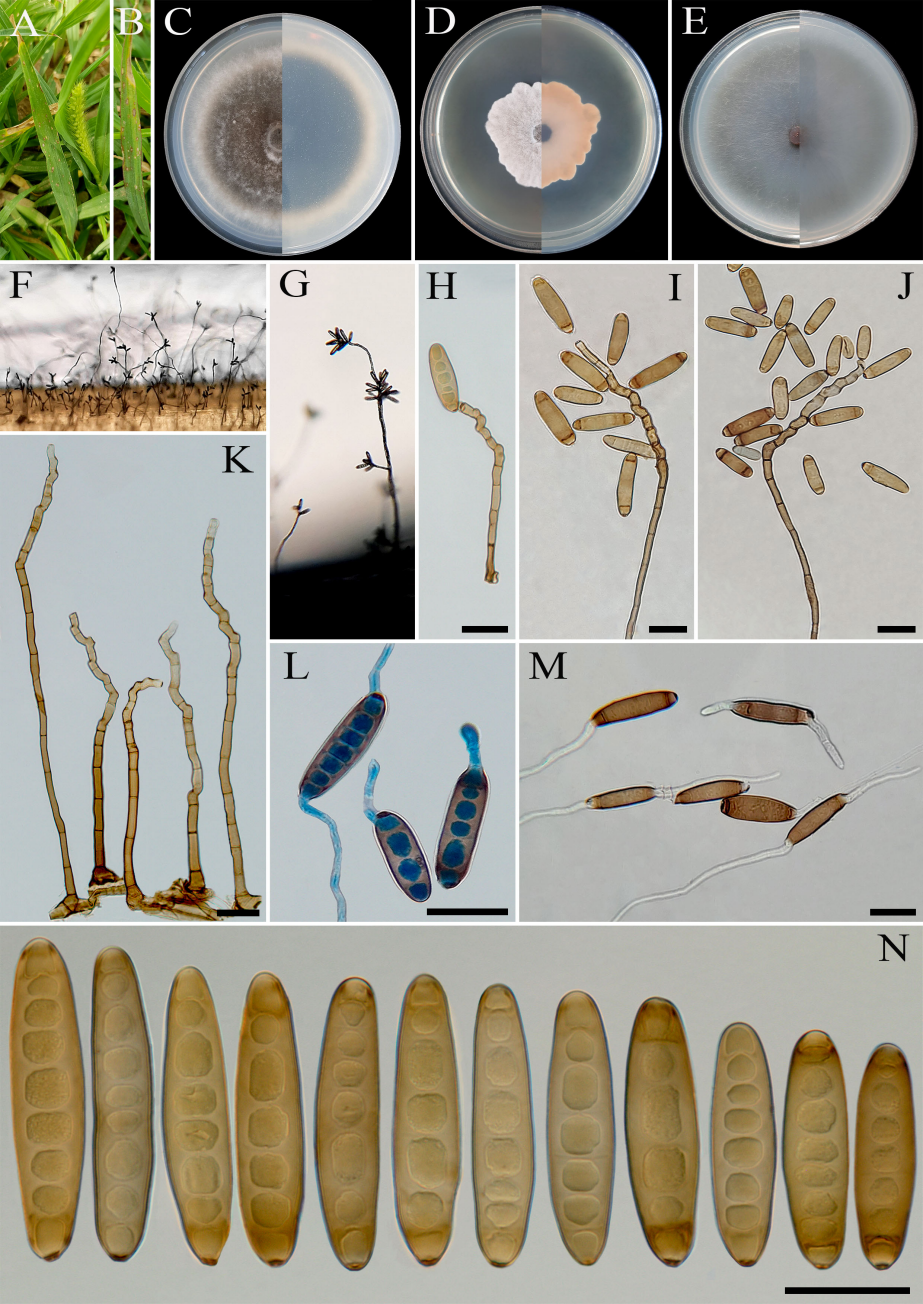
*Bipolaris banihashemii* (IRAN 3389C). **(A, B)** Lesions on host leaf (*Setaria* sp.). **(C−E)** Colonies (front and reverse) on PDA **(C)**, MEA **(D)**, and CMA **(E)** media after 7 days. **(F, G)** Sporulation pattern on TWA medium. **(H−K)** Conidiophores. **(L, M)** Germinated conidia. **(N)** Conidia. **(H−N)** Scale bars = 20 μm.

***Bipolaris banihashemii*** A. Ahmadpour, Z. Heidarian, Y. Ghosta, Z. Alavi & F. Alavi, sp. nov. (**Figure 6**).

MycoBank No: MB 854732

*Etymology*: Named in honor of Dr. Zia Banihashemi, emeritus Professor of Shiraz University, Iran, who significantly contributed to the knowledge of mycology and plant pathology in Iran.

*Diagnosis*: Differs from *Bipolaris variabilis* and *B. zeae* by the size of conidiophores and the shape and size of the conidia.

*Type*: IRAN, West Azarbaijan Province, Khoy County, on infected leaves of *Setaria* sp. (Poaceae, Poales), 20 September 2010, A. Ahmadpour, (IRAN 18244F, **holotype**, dried culture; **ex-type** IRAN 3389C).

*Description*: Leaf spots on *Setaria* sp., 1- to 5-mm long, gray at the center with red-brown margins. Sexual morph: Undetermined. Asexual morph: On TWA *Hyphae* 3- to 5-μm wide, pale brown to brown, smooth, septate, branched. *Conidiophores* (150–)260–400(–450) × 5–7 µm ( $\bar{x}$ ± SD = 330 ± 70 × 5 ± 1 μm, *n* = 50), mononematous, semi- to macronematous, arising singly or in groups, unbranched, straight to flexuous, septate, geniculate, pale brown to brown, paler toward the apex, swollen at the base. *Conidiogenous cells* (7–)9–23(–28) × 5–8 μm ( $\bar{x}$ ± SD = 16 ± 7 × 6.5 ± 1.5 μm, *n* = 50), mono- to polytretic, sympodial proliferation, integrated, terminal or intercalary, subcylindrical to slightly swollen, pale brown to brown, smooth walled to slightly verruculose, with thickened and darkened scars. *Conidia* (28–)38–62(–68) × 9–13 µm ( $\bar{x}$ ± SD = 50 ± 18.5 × 11 ± 2 μm, *n* = 50), golden brown, smooth walled, straight, cylindrical to fusoid, occasionally ellipsoidal, tapering toward rounded ends, end cells often cut off by a thick dark septum, (4–)5–8(–9)-distoseptate, germination mono- or bipolar; hila 2- to 3-μm wide, truncate, slightly protruding, thickened, and darkened. *Stroma*, *chlamydospores*, and *microconidiation* were not observed.

*Culture characteristics*: Colonies on PDA reaching 67 mm in diameter after 7 days at 25°C in the dark, circular, margin entire, olivaceous green at the center, white at the margin with white to gray aerial mycelia; reverse gray olivaceous to olivaceous black with a hyaline margin. Colonies on MEA reaching 35 mm in diameter, circular, margin irregular, cottony appearance, white with white aerial mycelia; reverse brown to pale brown from the center to the margin. Colonies on CMA reaching 62 mm in diameter, circular, margin entire, hairy appearance, olivaceous gray with sparse white to gray aerial mycelia; reverse olivaceous gray at the center and a hyaline margin.

*Additional materials examined*: IRAN, West Azarbaijan Province, Khoy County, on infected leaves of *Setaria* sp. (Poaceae, Poales), 20 September 2010, A. Ahmadpour, isolate IRAN 3388C; *ibid.* on infected leaves of *Setaria* sp. (Poaceae, Poales), 20 September 2010, A. Ahmadpour, isolate IRAN 3387C.

*Host and distribution*: *Setaria* sp. in Iran (this study).

**Figure 7:**
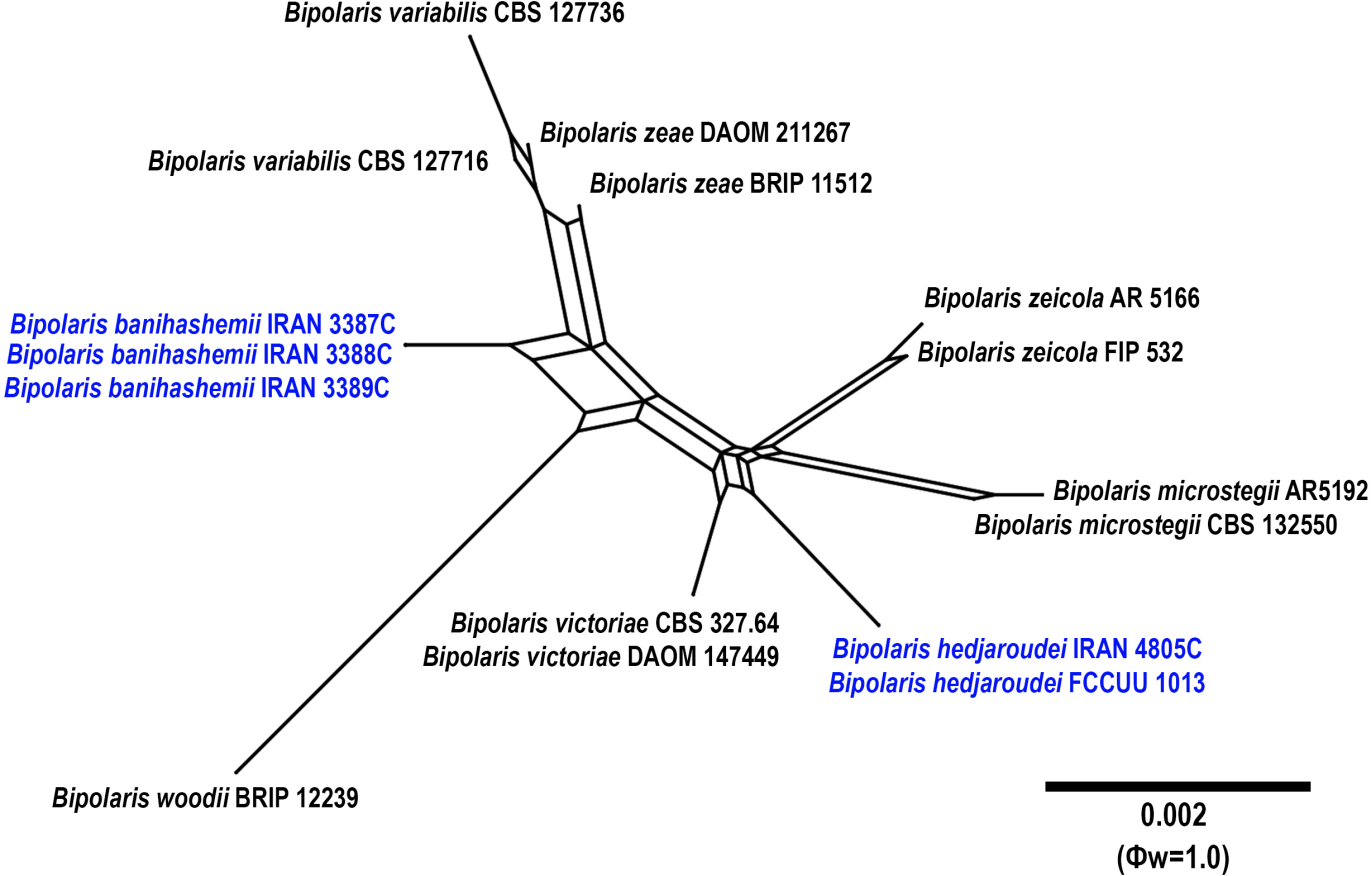
Split graphs showing the results of PHI test of *Bipolaris banihashemii* and *B. hedjaroudei* with their most closely related species (Φw = 1.0). The new taxa are shown in bold blue.

*Notes:* Based on multi-locus phylogenetic analyses, *B. banihashemii* clustered closely with *B. variabilis* and *B. zeae* (MLBS/MPBS/BIPP = 100/86/0.99) (**Figure 1**). A comparison of nucleotide differences in ITS−rDNA, *GAPDH*, and *TEF1* indicates that *B. banihashemii* (IRAN 3389C) differs from *B. variabilis* (CBS 127716) by 1/548 bp [0.18%, with one gap (0%)] in ITS−rDNA, 4/577 bp (0.69%) in *GAPDH*, and 1/642 bp (0.15%) in *TEF1* and from *B. zeae* (BRIP 11512) by 3/577 bp (0.52%) in *GAPDH* and 2/712 bp (0.28%) in *TEF1*. The PHI analysis confirms that *B. banihashemii* has no significant genetic recombination with closely related species (Φw = > 0.05, **Figure 7**). *Bipolaris variabilis* can be differentiated by having longer conidiophores (up to 1,600 μm *vs.* up to 450 μm in *B. banihashemii*), shape of conidia (verruculose walled, straight or slightly curved, globose to obclavate conidia *vs.* smooth walled, straight, cylindrical to fusoid conidia in *B. banihashemii*), and wider conidia (10–19.5 *vs.* 9–13 μm in *B. banihashemii*) (Marin-Felix et al., 2017a). *Bipolaris zeae* differs from *B. banihashemii* in producing shorter conidiophores (up to 370 *vs.* 450 μm in *B. banihashemii*) and longer and wider conidia [(30–)40–80(–120) × 12–18(–21) μm *vs.* (28–)38–62(–68) × 9–13 µm in *B. banihashemii*] (Sivanesan, 1987; Manamgoda et al., 2014).

**Figure 8:**
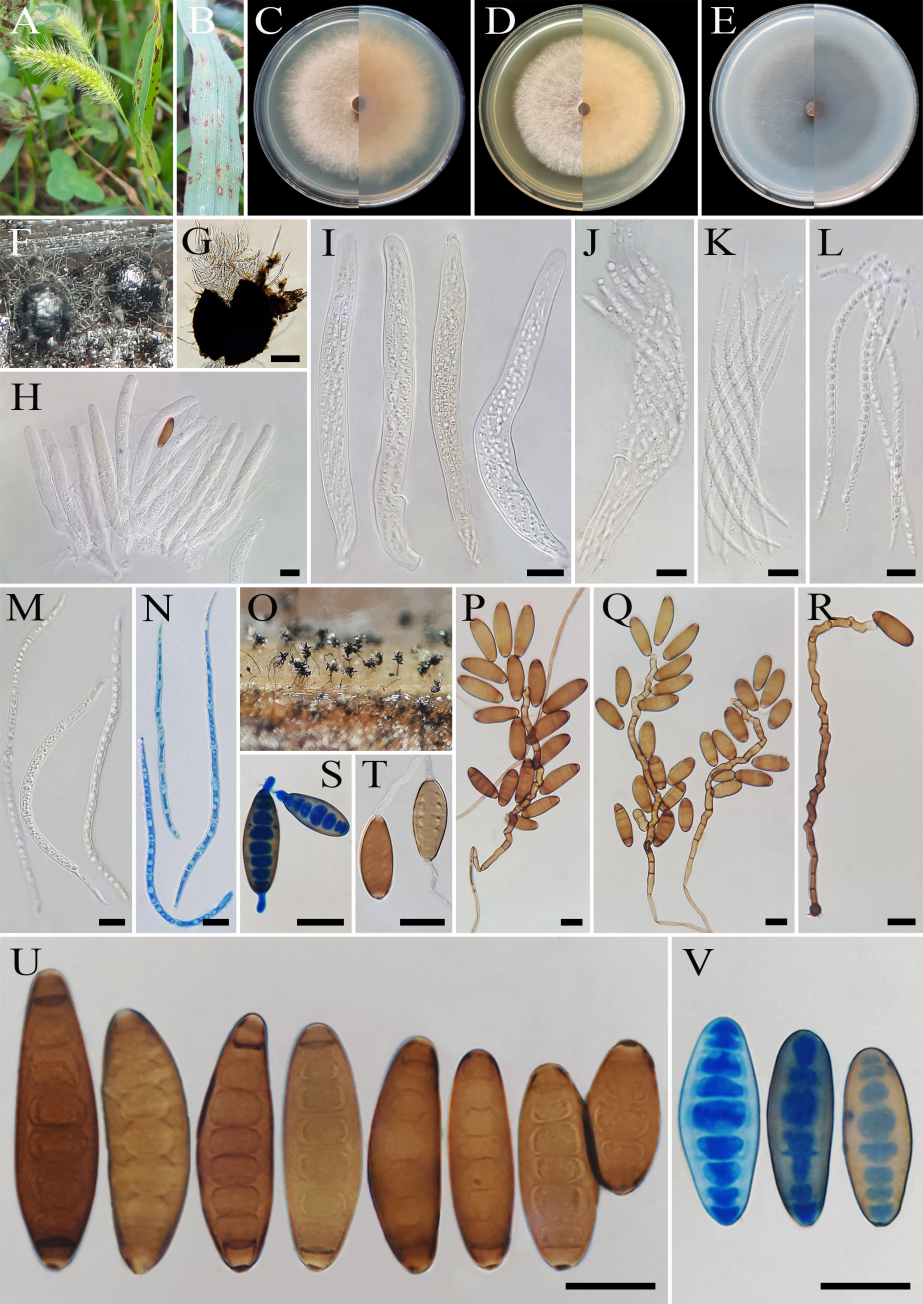
*Bipolaris hedjaroudei* (IRAN 4805C). **(A, B)** Lesions on host leaf (*Setaria* sp.). **(C−E)** Colonies (front and reverse) on PDA **(C)**, MEA **(D)**, and CMA **(E)** media after 7 days. **(F, G)** Ascomata on TWA medium containing leaves of the host plant. **(H−N)** Asci and ascospores. **(O)** Sporulation pattern on TWA medium. **(P–R)** Conidiophores. **(S, T)** Germinated conidia. **(U, V)** Conidia. **(G)** Scale bars = 100 μm. **(H–V)** Scale bars = 20 μm.

***Bipolaris hedjaroudei*** A. Ahmadpour, Z. Heidarian, Y. Ghosta, Z. Alavi & F. Alavi, sp. nov. (**Figure 8**).

MycoBank No: MB 854733

*Etymology*: Named in honor of Dr. Ghorbanali Hedjaroud, emeritus Professor of Tehran University, who significantly contributed to the knowledge of mycology in Iran.

*Diagnosis*: Differs from *Bipolaris microstegii*, *B. victoriae*, *B. zeicola*, and *B. woodii* by having longer conidiophores, smaller conidia, and production of sexual morph (homothallic species) in culture media.

*Type*: IRAN, West Azarbaijan Province, Khoy County, on infected leaves of *Setaria* sp. (Poaceae, Poales), 10 September 2020, A. Ahmadpour, (IRAN 18492F, **holotype**, dried culture; **ex-type** IRAN 4805C).

*Description*: On infected leaves of *Setaria* sp., leaf lesions 1- to 10-mm long, gray at the center and red-brown at the margins. Sexual morph: On sterile leaves of *Setaria* sp. in TWA medium *Ascomata* pseudothecial, (300–)400–550(–600) × (290–)300–500(–550) μm ( $\bar{x}$ ± SD = 475 ± 75 × 400 ± 100 μm, *n* = 20), solitary, scattered, superficial or slightly embedded, globose to subglobose or oval, dark brown to black, unilocular with a short ostiolate neck, with long brown setae and conidiophores bearing conidia developing on the upper part of the ascoma. *Ostiolar neck* 10–20× 8–12 μm ( $\bar{x}$ ± SD = 15 ± 5 × 10 ± 2 μm, *n* = 20), conical, central, filled with masses of hyaline cells frequently covering the apex of the neck. *Peridium* (ascomata wall) 30- to 35-μm wide, composed of layers of pigmented thick-walled cells. *Pseudoparaphyses* 2- to 3-μm wide, hyaline, septate, filamentous, simple to branched. *Asci* (136–)150–200(–212) × (15–)17–20 (–22) μm ( $\bar{x}$ ± SD = 150 ± 50 × 18.5 ± 1.5 μm, *n* = 20), with eight ascospores coiled in a tight helix, bitunicate, cylindrical to clavate, occasionally obclavate–fusoid, straight or curved, with short pedicel. *Ascospores* 170–250 × 5–7 μm ( $\bar{x}$ ± SD = 210± 40 × 6 ± 1 μm, *n* = 50), hyaline, filiform to flagelliform, tapering toward the rounded ends, tightly coiled inside the ascus, 7–13 septate, with a thin mucilaginous sheath visible in water mounts. Asexual morph: On TWA *Hyphae* 2- to 5-μm wide, pale brown to brown, smooth, septate, branched. *Conidiophores* (125–)175–250(–325) × 5–7 µm ( $\bar{x}$ ± SD = 212.5 ± 37.5 × 6 ± 1 μm, *n* = 50), mononematous, semi- to macronematous, arising mostly singly or rarely in groups, unbranched, straight to flexuous, septate, geniculate, pale brown to brown, paler toward the apex, swollen at the base. *Conidiogenous cells* (6–)8–22(–25) × 5–8 μm ( $\bar{x}$ ± SD = 15 ± 7 × 6.5 ± 1.5 μm, *n* = 50), mono- to polytretic, sympodial proliferation, integrated, terminal or intercalary, subcylindrical to slightly swollen, pale brown to brown, smooth walled to slightly verruculose, with thickened and darkened scars. *Conidia* (25–)32–60(–62) × 15–17 µm ( $\bar{x}$ ± SD = 46 ± 14 × 16 ± 1 μm, *n* = 50), brown to dark brown, smooth walled, straight to slightly curved, broadly fusiform, occasionally ellipsoidal to obclavate, tapering toward the rounded ends, apical and basal cells paler than the median cells, end cells often cut off by a thick dark septum, (4–)5–8(–9)-distoseptate, germinated mono- or bipolar; hila 2- to 3-μm wide, conspicuous, brown, slightly protuberant, thickened, and darkened. *Stroma*, *chlamydospores*, and *microconidiation* were not observed.

*Culture characteristics*: Colonies on PDA reaching 65 mm in diameter after 7 days at 25°C in the dark, circular, margin entire, velvety, gray at the center and white at the margin, with gray to white aerial mycelia; reverse brown to pale brown from the center to the margin. Colonies on MEA reaching 61 mm in diameter, circular, margin entire, cottony appearance, white with white aerial mycelia; reverse brown to pale brown from the center to the margin. Colonies on CMA reaching 61 mm in diameter, circular, margin entire, hairy appearance, gray with sparse white to gray aerial mycelia; reverse olivaceous brown at the center and a hyaline margin.

*Additional material examined*: Iran, West Azarbaijan Province, Khoy County, on infected leaves of *Setaria* sp. (Poaceae, Poales), 10 September 2020, A. Ahmadpour, isolate FCCUU 1013.

*Host and distribution*: *Setaria* sp. in Iran (this study).

*Notes: Bipolaris hedjaroudei* is phylogenetically closely related to *B. victoriae*, *B. microstegii*, *B. zeicola*, and *B. woodii* (**Figure 1**). Pairwise sequence similarity analyses of three genomic regions in *B. hedjaroudei* distinguished it from closely related taxa. A comparison of nucleotide differences in ITS−rDNA, *GAPDH*, and *TEF1* indicates that *B. hedjaroudei* (IRAN 4805C) differs from *B. microstegii* (CBS 132550) by 2/507 bp (0.39%) in ITS−rDNA, 5/539 bp (0.92%) in *GAPDH*, and 8/763 bp [1.04%, with two gaps (0%)] in *TEF1*; from *B. victoriae* (CBS 327.64) by 1/480 bp (0.20%) in *GAPDH* and 6/763 bp [0.78%, with two gaps (0%)] in *TEF1*; from *B. woodii* (BRIP 12239) by 2/512 bp (0.39%) in ITS−rDNA, 9/550 bp [1.63%, with one gap (0%)] in *GAPDH*, and 8/761 bp [1.05%, with two gaps (0%)] in *TEF1*; and from *B. zeicola* (FIP 532) by 2/437 bp [0.45%, with one gap (0%)] in ITS−rDNA, 1/480 bp (0.20%) in *GAPDH*, and 6/763 bp [0.78%, with two gaps (0%)] in *TEF1*. The PHI analysis confirms that *B. hedjaroudei* has no significant genetic recombination with closely related species (Φw = > 0.05, **Figure 7**). *Bipolaris hedjaroudei* can be differentiated by having longer conidiophores (up to 325 μm *vs.* up to 250 μm in *B. victoriae*, up to 270 μm in *B. zeicola*, up to 250 μm in *B. woodii*) and smaller conidia [(25–)32–60(–62) × 15–17 µm *vs.* (25–)55–90(–110) × (10–)12–16(–19) μm in *B. victoriae*, (45–)65–90(–105) × (10–)15–19(–22) μm in *B. zeicola*, (60–)69–76(–86) × (10–)12.5–13.5(–15) μm in *B. woodii*] (Manamgoda et al., 2014; Tan et al., 2016). *Bipolaris microstegii* differs from *B. hedjaroudei* in producing secondary conidiophores and conidia, longer conidiophores (up to 750 μm *vs.* up to 325 μm in *B. hedjaroudei*), and accentuated septa (Manamgoda et al., 2014). *Bipolaris victoriae* and *B. zeicola* have been reported on various poaceous hosts and caused destructive diseases in oat and maize, respectively (Manamgoda et al., 2014; Farr et al., 2024). *Bipolaris hedjaroudei* is a homothallic species that forms sexual morph abundantly on TWA medium containing host leaves after 21–30 days. In contrast, *B. victoriae* and *B. zeicola* are heterothallic species, and the sexual morph of *B. microstegii* and *B. woodii* has not been recorded yet (Manamgoda et al., 2014; Tan et al., 2016).

**Figure 9:**
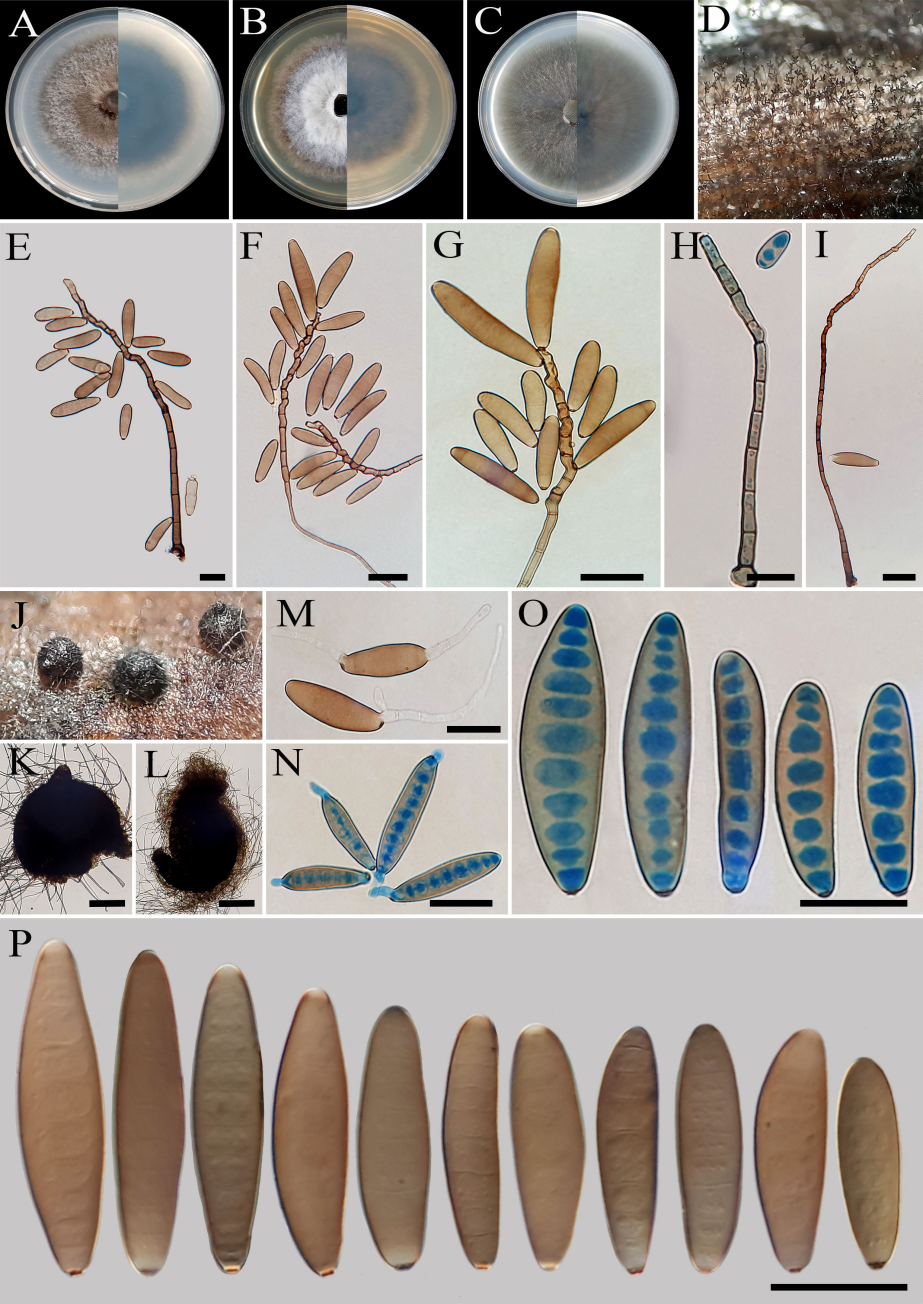
*Bipolaris hemerocallidis* (IRAN 4774C). **(A−C)** Colonies (front and reverse) on PDA **(A)**, MEA **(B)**, and CMA **(C)** media after 7 days. **(D)** Sporulation pattern on TWA medium. **(E–I)** Conidiophores. **(J−L)** Sterile ascomata on TWA medium containing leaves of the host plant. **(M, N)** Germinated conidia. **(O, P)** Conidia. **(K, L)** Scale bars = 50 μm, **(E–I)**, **(M–P)** Scale bars = 20 μm.

***Bipolaris hemerocallidis*** A. Ahmadpour, Z. Heidarian, Y. Ghosta, Z. Alavi & F. Alavi, sp. nov. (**Figure 9**).

MycoBank No: MB 854734

*Etymology*: Named after the host genus, *Hemerocallis*, from which the holotype was collected.

*Diagnosis*: Differs from *Bipolaris axonopicola* by having longer conidiophores.

*Type*: IRAN, Isfahan Province, Isfahan County, Flower Garden, on leaves of *Hemerocallis fulva* (Asphodelaceae, Asparagales), 7 October 2013, A. Ahmadpour/Z. Heidarian, (IRAN 18206F, **holotype**, dried culture; **ex–type** IRAN 4774C).

*Description*: Associated with leaves of *Hemerocallis fulva*. Sexual morph: Undetermined. Asexual morph: On TWA *Hyphae* 2- to 5-μm wide, pale brown to brown, smooth, septate, branched. *Conidiophores* (180–)230–550(–600) × 5–7 µm ( $\bar{x}$ ± SD = 390 ± 160 × 6 ± 1 μm, *n* = 50), mononematous, semi- to macronematous, arising singly or in groups, unbranched, straight to flexuous, septate, geniculate, pale brown to brown, paler toward the apex, swollen at the base. *Conidiogenous cells* (5–)7–21(–25) × 5–8 μm ( $\bar{x}$ ± SD = 14± 7 × 6.5 ± 1.5 μm, *n* = 50), mono- to polytretic, sympodial proliferation, integrated, terminal or intercalary, subcylindrical to slightly swollen, pale brown to brown, smooth walled to slightly verruculose, with thickened and darkened scars. *Conidia* (38–)40–52(–60) × 9–11 µm ( $\bar{x}$ ± SD = 46 ± 6 × 10 ± 1 μm, *n* = 50), pale brown to brown, smooth walled, straight to slightly curved, broadly fusoid to cylindrical, occasionally ellipsoidal to clavate, tapering toward the rounded ends, apical and basal cells paler than the median cells, (4–)5–9(–10)-distoseptate, germinated mono- or bipolar; hila 2- to 3-μm wide, truncate, slightly protruding, thickened, and darkened. *Stroma*, *chlamydospores*, and *microconidiation* were not observed.

*Culture characteristics*: Colonies on PDA reaching 65 mm in diameter after 7 days at 25°C in the dark, circular, margin entire, olivaceous green at the center with white to gray aerial mycelia, white at the margin; reverse olivaceous gray to olivaceous black. Colonies on MEA reaching 55 mm in diameter, circular, margin entire, cottony appearance, olivaceous gray to gray, with white to gray aerial mycelia; reverse brown to pale brown from the center to the margin. Colonies on CMA reaching 60 mm in diameter, circular, margin entire, hairy appearance, olivaceous gray with sparse white to gray aerial mycelia; reverse olivaceous brown at the center with a hyaline margin. Sterile ascomata were produced on TWA medium containing leaves of the host plant. However, these structures remained sterile (without asci and ascospores) after 3–6 months of incubation.

*Additional material examined*: IRAN, Isfahan Province, Isfahan County, Flower Garden, on leaves of *Hemerocallis fulva* (Asphodelaceae, Asparagales), 7 October 2013, A. Ahmadpour/Z. Heidarian, isolate FCCUU 1011.

*Host and distribution*: Associated with leaves of *Hemerocallis fulva* in Iran (this study).

*Notes: Bipolaris hemerocallidis* is phylogenetically close to *B. axonopicola* (MLBS/MPBS/BIPP = 100/100/1.0) (**Figure 1**). The pairwise DNA sequence comparison revealed that *B. hemerocallidis* is distinct from *B. axonopicola*. A comparison of nucleotide differences in ITS−rDNA, *GAPDH*, and *TEF1* indicates that *B. hemerocallidis* (IRAN 4774C) differs from *B. axonopicola* (BRIP 11740) by 6/532 bp [1.12%, with four gaps (0%)] in ITS−rDNA, 17/546 bp (3.11%) in *GAPDH* and 4/788 bp (0.50%) in *TEF1*. The PHI analysis confirms that *B. hemerocallidis* has no significant genetic recombination with closely related species (Φw = > 0.05, **Figure 5**). *Bipolaris hemerocallidis* can be differentiated by having longer conidiophores (up to 600 μm *vs.* up to 250 μm in *B. axonopicola*) (Tan et al., 2016). *Bipolaris axonopicola* is only known on *Axonopus fissifolius* (Poaceae) in Australia (Tan et al., 2016). In this study, *B. hemerocallidis* was isolated from the leaves of *Hemerocallis fulva* (Asphodelaceae, Asparagales) in the greenhouse.

**Figure 10:**
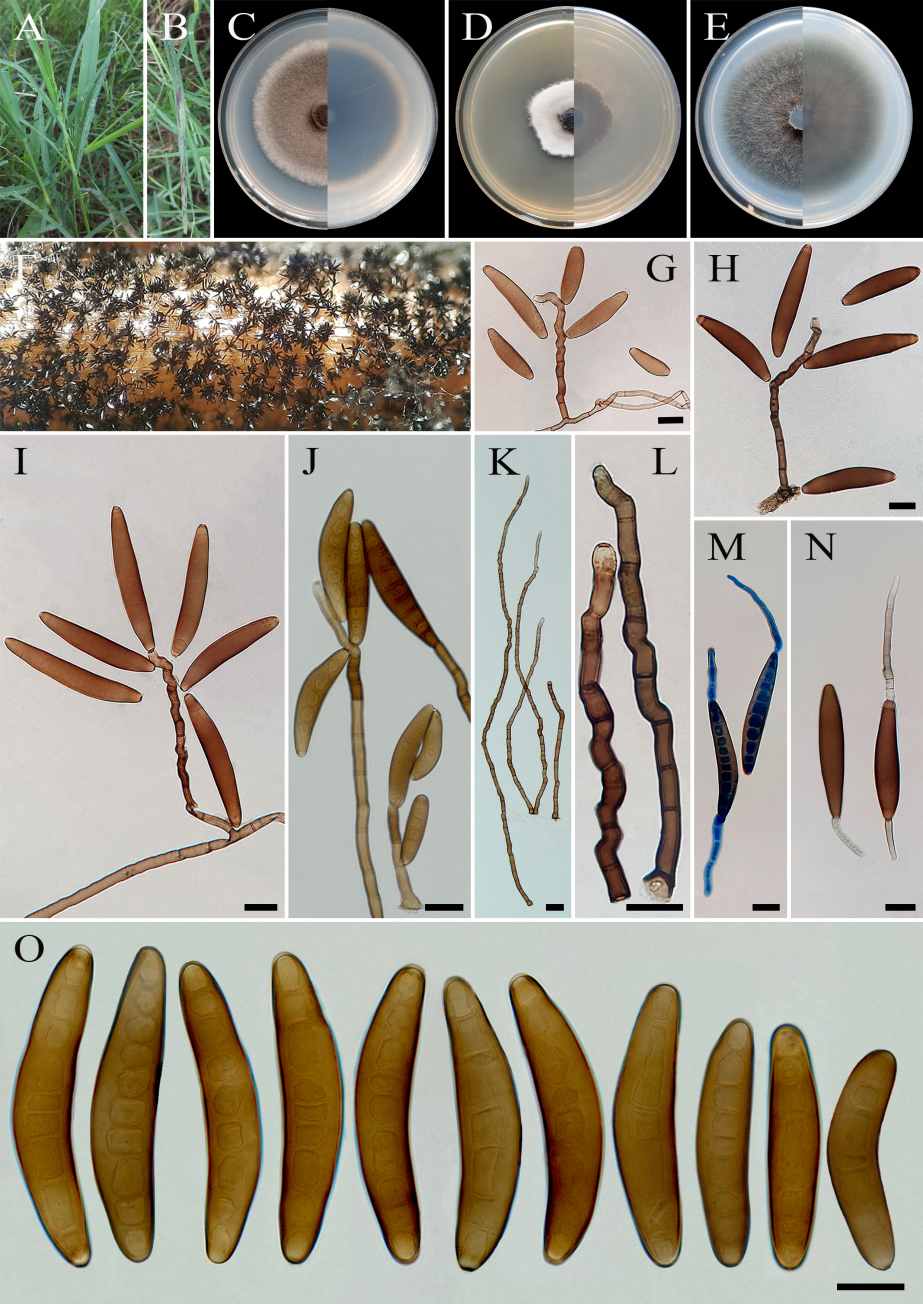
*Bipolaris iranica* (IRAN 4775C). **(A, B)** Lesions on host leaf (*Cynodon dactylon*). **(C−E)** Colonies (front and reverse) on PDA **(C)**, MEA **(D)**, and CMA **(E)** media after 7 days. **(F)** Sporulation pattern on TWA medium. **(G–L)** Conidiophores. **(M, N)** Germinated conidia. **(O)** Conidia. **(J−O)** Scale bars = 20 μm.

***Bipolaris iranica*** A. Ahmadpour, Z. Heidarian, Y. Ghosta, Z. Alavi & F. Alavi, sp. nov. (**Figure 10**).

MycoBank No: MB 854735

*Etymology*: Named after the country “Iran” where the holotype was collected.

*Diagnosis*: Differs from *Bipolaris heveae*, *B. microlaenae*, and *B. simmondsii* by having much longer conidiophores and accentuated transverse septa.

*Type*: IRAN, West Azarbaijan Province, Khoy County, on infected leaves of *Cynodon dactylon* (Poaceae, Poales), 20 September 2010, A. Ahmadpour, (IRAN 18207F, **holotype**, dried culture; **ex–type** IRAN 4775C).

*Description*: Leaf lesions on *Arundo donax*, *Cynodon dactylon*, *Echinochloa colona*, *Hordeum vulgare*, *Sorghum halepense*, and *Triticum aestivum*, 1- to 10-mm long. Sexual morph: Undetermined. Asexual morph: On TWA *Hyphae* 2- to 5-μm wide, pale brown to brown, smooth, septate, branched. *Conidiophores* (125–)187–480(–550) × 7–8 µm ( $\bar{x}$ ± SD = 337.5 ± 146.5 × 7.5 ± 0.5 μm, *n* = 50), mononematous, macronematous, arising singly or in groups, simple, straight to flexuous, septate, geniculate, with cell walls thicker than vegetative hyphae, pale brown to brown, paler toward the apex, basal cell swollen and darker than the other cells, up to 10 μm in diameter *Conidiogenous cells* (8–)10–24(–28) × 6–8 μm ( $\bar{x}$ ± SD = 17 ± 7 × 7 ± 1 μm, *n* = 50), mono- to polytretic, proliferating sympodially, integrated, terminal or intercalary, subcylindrical to slightly swollen, pale brown to dark brown, smooth walled to slightly verruculose, with thickened and darkened scars. *Conidia* (70–)85–100(–110)× 15–20 µm ( $\bar{x}$ ± SD = 92.5 ± 7.5 × 17.5 ± 2.5 μm, *n* = 50), brown to dark brown, smooth walled, straight to curved, mostly navicular to fusoid, rarely cylindrical to clavate, taper toward rounded ends, apical and basal cells paler than the median cells, septa accentuated at maturity, (6–)8–11(–13)-distoseptate, germinated mono- or bipolar; hila 2- to 3-μm wide, flat to slightly protuberant, thickened, and darkened. *Stroma*, *chlamydospores*, and *microconidiation* were not observed.

*Culture characteristics*: Colonies on PDA reaching 58 mm in diameter after 7 days at 25°C in the dark, circular, margin entire, olivaceous gray at the center with white to gray aerial mycelia, white at the margin; reverse olivaceous gray to olivaceous black with a hyaline margin. Colonies on MEA reaching 42 mm in diameter, circular, margin entire, cottony appearance, white with white aerial mycelia; reverse brown to pale brown from the center to the margin. Colonies on CMA reaching 66 mm in diameter, circular, margin entire, olivaceous gray with sparse gray aerial mycelia; reverse olivaceous gray at the center and a hyaline margin.

*Additional materials examined*: IRAN, West Azarbaijan Province, Miyandoab County, on infected leaves of *Sorghum halepense* (Poaceae, Poales), 11 July 2013, A. Ahmadpour/Z. Heidarian, isolate FCCUU 1005; *ibid.* on infected leaves of *Echinochloa colona* (Poaceae, Poales), 23 September 2014, A. Ahmadpour/Z. Heidarian, isolate FCCUU 1007; West Azarbaijan Province, Urmia County, on infected leaves of *Arundo donax* (Poaceae, Poales), 20 September 2014, A. Ahmadpour/Z. Heidarian, isolate FCCUU 1006; West Azarbaijan Province, Bukan County, on infected leaves of *Hordeum vulgare* (Poaceae, Poales), 20 October 2014, A. Ahmadpour/Z. Heidarian, isolate FCCUU 1008; West Azarbaijan Province, Khoy County, on infected leaves of *Triticum aestivum* (Poaceae, Poales), 22 May 2021, A. Ahmadpour, isolate FCCUU 1009.

*Hosts and distribution*: *Arundo donax*, *Cynodon dactylon*, *Echinochloa colona*, *Hordeum vulgare*, *Sorghum halepense*, and *Triticum aestivum* in Iran (this study).

**Figure 11:**
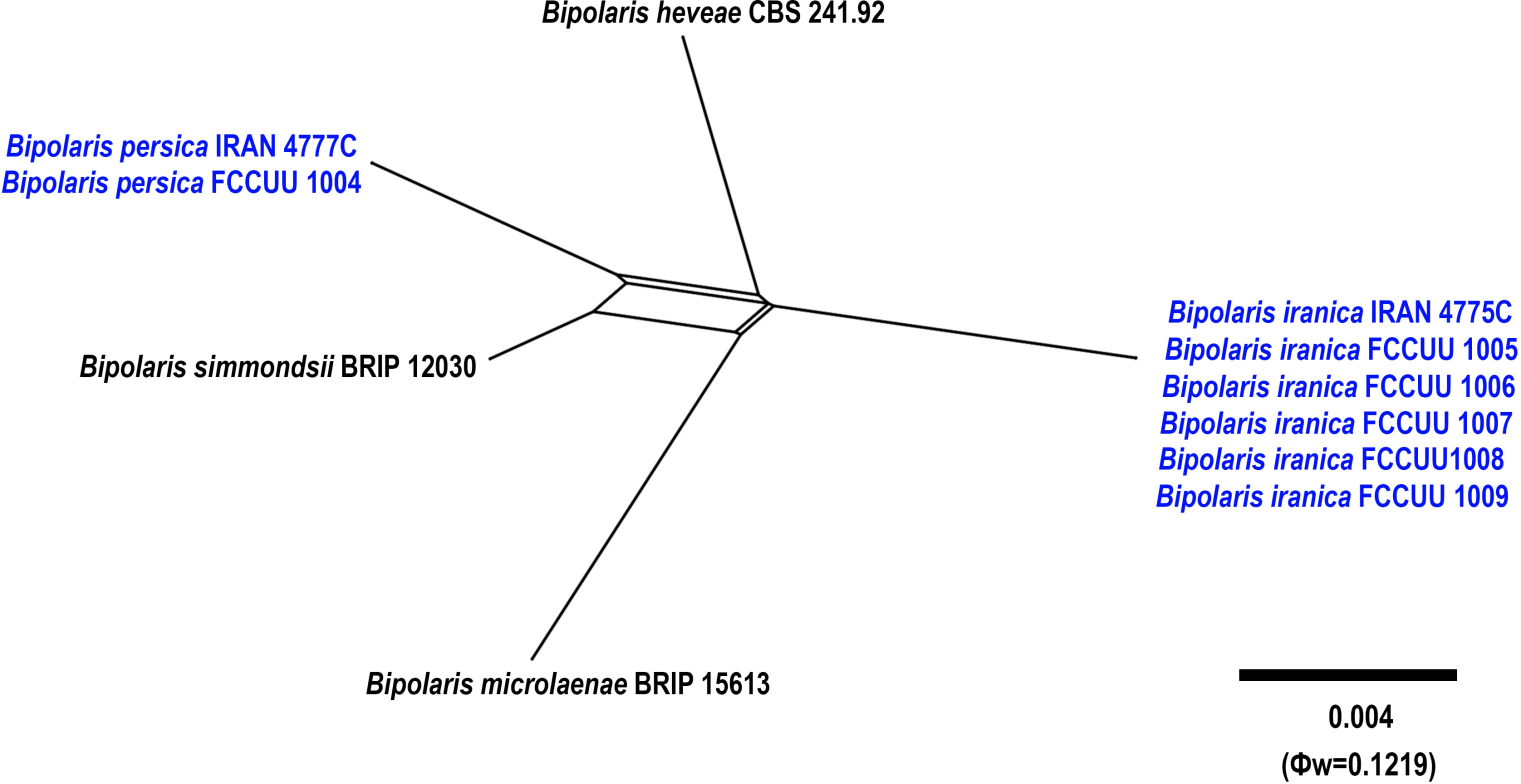
Split graphs showing the results of PHI test of *Bipolaris iranica* and *B. persica* with their most closely related species (Φw = 0.1219). The new taxa are shown in bold blue.

*Notes:* Based on the results of phylogenetic analyses (**Figure 1**), *B. iranica* isolates clustered well in a separate lineage with 100% ML, 100% MP bootstrap, and 1.0 BI posterior probability values, representing a new taxon. The pairwise DNA sequence comparison revealed that *B. iranica* is distinct from related taxa, *B. heveae*, *B. microlaenae*, and *B. simmondsii*. A comparison of nucleotide differences in ITS−rDNA, *GAPDH*, and *TEF1* indicates that *B. iranica* (IRAN 4775C) differs from *B. heveae* (CBS 241.92) by 5/539 bp [0.92%, with four gaps (0%)] in ITS−rDNA, 11/490 bp (2.24%) in *GAPDH* and 6/770 bp (0.77%) in *TEF1*; from *B. microlaenae* (BRIP 15613) by 2/534 bp (0.37%) in ITS−rDNA, 11/543 bp (2.02%) in *GAPDH*, and 12/788 bp (1.52%) in *TEF1*; and from *B. simmondsii* (BRIP 12030) by 3/536 bp [0.55%, with one gap (0%)] in ITS−rDNA, 13/536 bp (2.42%) in *GAPDH*, and 7/788 bp (0.88%) in *TEF1*. The PHI analysis confirms that *B. iranica* has no significant genetic recombination with closely related species (Φw = > 0.05, **Figure 11**). *Bipolaris iranica* is morphologically similar to *B. heveae*, *B. microlaenae*, and *B. simmondsii*; however, it can be distinguished by its much longer conidiophores (up to 550 μm *vs.* up to 335 μm in *B. heveae*, and up to 240 μm in *B. simmondsii*), and its accentuated septa, which are absent in *B. heveae*, *B. microlaenae*, and *B. simmondsii* (Manamgoda et al., 2014; Tan et al., 2016). *Bipolaris heveae* is known to cause diseases on rubber trees (*Hevea brasiliensis*) across various tropical countries, including Cambodia, the Dominican Republic, Ghana, Guatemala, Haiti, Honduras, Indonesia, Mexico, Nigeria, the Philippines, Sri Lanka, and the United States. Unlike *B. iranica*, this pathogen does not infect grass species and is restricted to its specific host plant (Manamgoda et al., 2014; Farr et al., 2024). Furthermore, *B. microlaenae* and *B. simmondsii* have only been documented in Australia, where they cause leaf spots on *Zoysia macrantha* (Tan et al., 2016).

**Figure 12:**
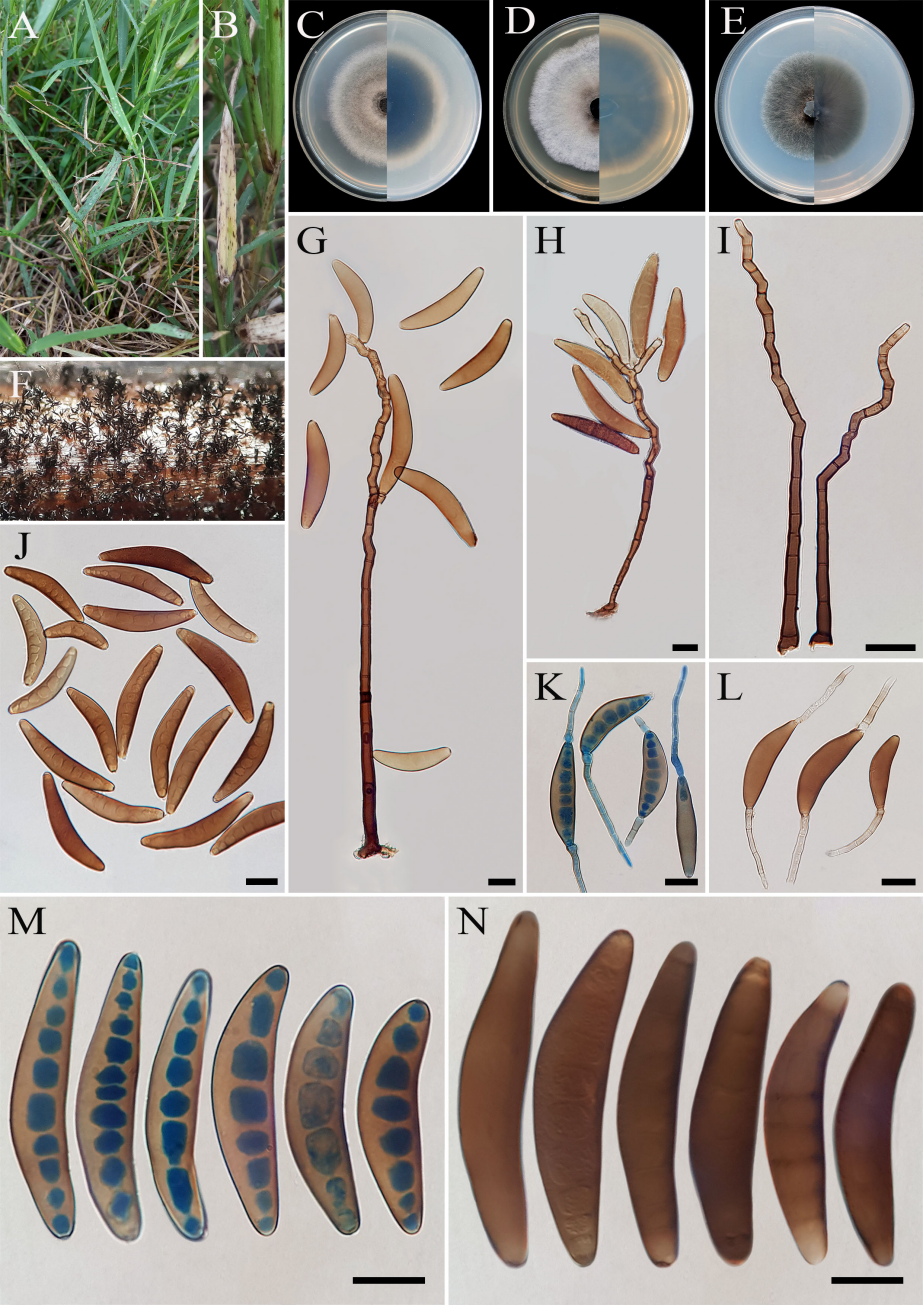
*Bipolaris persica* (IRAN 4777C). **(A, B)** Lesions on host leaf (*Cynodon dactylon*). **(C−E)** Colonies (front and reverse) on PDA **(C)**, MEA **(D)**, and CMA **(E)** media after seven days. **(F)** Sporulation pattern on TWA medium. **(G–I)** Conidiophores. **(K, L)** Germinated conidia. **(J**, **M, N)** Conidia. **(G−N)** Scale bars = 20 μm.

***Bipolaris persica*** A. Ahmadpour, Z. Heidarian, Y. Ghosta, Z. Alavi & F. Alavi, sp. nov. (**Figure 12**)

MycoBank No: MB 854736

*Etymology*: The name refers to the old name of Iran, Persia, from where the holotype was collected.

*Diagnosis*: Differs from *Bipolaris heveae*, *B. microlaenae*, and *B. simmondsii* by having longer conidiophores and accentuated transverse septa.

*Type*: IRAN, West Azarbaijan Province, Mahabad County, on infected leaves of *Cynodon dactylon* (Poaceae, Poales), 20 June 2015, A. Ahmadpour, (IRAN 18209F, **holotype**, dried culture; **ex-type** IRAN 4777C).

*Description*: Leaf spots on *Cynodon dactylon*, 1- to 10-mm long, with dark brown spots. Sexual morph: Undetermined. Asexual morph: On TWA *Hyphae* 2- to 5-μm wide, pale brown to brown, smooth, septate, branched. *Conidiophores* (210–)250–330(–350) × 6–7 µm ( $\bar{x}$ ± SD = 290 ± 40 × 6.5 ± 0.5 μm, *n* = 50), mononematous, macronematous, arising singly or in groups, simple, straight to flexuous, septate, geniculate, with cells wall thicker than those of vegetative hyphae, pale brown to brown, paler toward the apex, basal cell swollen and darker than the other cells, up to 10 μm in diameter. *Conidiogenous cells* (7–)10–25(–30) × 6–8 μm ( $\bar{x}$ ± SD = 17.5 ± 7.5 × 7 ± 1 μm, *n* = 50), mono- to polytretic, proliferating sympodially, integrated, terminal or intercalary, subcylindrical to slightly swollen, pale brown to brown, smooth walled to slightly verruculose, with thickened and darkened scars. *Conidia* (62–)80–95(–100)× 13–20 µm ( $\bar{x}$ ± SD = 87.5 ± 7.5 × 16.5 ± 3.5 μm, *n* = 50), brown to dark brown, smooth walled, curved, mostly navicular to fusoid, tapering toward the rounded ends, apical and basal cells paler than the median cells, septa accentuated at maturity, (6–)7–10(–11)-distoseptate, germinated mono- or bipolar; hila 2- to 3-μm wide, flat, thickened, and darkened. *Stroma*, *chlamydospores*, and *microconidiation* were not observed.

*Culture characteristics*: Colonies on PDA reaching 68 mm in diameter after 7 days at 25°C in the dark, circular, margin entire, olivaceous gray with white to gray aerial mycelia, sporulation abundant; reverse olivaceous gray at the center with hyaline margin. Colonies on MEA reaching 58 mm in diameter, circular, margin entire, cottony appearance, gray at the center, white at the margin with white to gray aerial mycelia; reverse olivaceous gray with hyaline margin. Colonies on CMA reaching 53 mm in diameter, circular, margin entire, olivaceous green with white to gray aerial mycelia; reverse olivaceous gray to olivaceous black and a hyaline margin.

*Additional material examined*: IRAN, West Azarbaijan Province, Mahabad County, on infected leaves of *Cynodon dactylon* (Poaceae, Poales), 20 June 2015, A. Ahmadpour, isolate FCCUU 1004.

*Host and distribution*: *Cynodon dactylon* in Iran (this study).

*Notes: Bipolaris persica* is phylogenetically closely related to *B. heveae*, *B. iranica*, *B. microlaenae*, and *B. simmondsii* (**Figures 1**, **11**). Pairwise DNA sequence comparison revealed that *B. persica* is distinct from its closely related taxa. A comparison of nucleotide differences in ITS−rDNA, *GAPDH*, and *TEF1* indicates that *B. persica* (IRAN 4777C) differs from *B. heveae* (CBS 241.92) by 1/500 bp [0.20%, with one gap (0%)] in ITS−rDNA, 13/481 bp (2.70%) in *GAPDH*, and 7/731 bp (0.95%) in *TEF1*; from *B. iranica* (IRAN 4775C) by 4/500 bp [0.80%, with three gaps (0%)] in ITS−rDNA, 15/504 bp (2.97%) in *GAPDH*, and 8/744 bp (1.07%) in *TEF1*; from *B. microlaenae* (BRIP 15613) by 4/500 bp [0.80%, with three gaps (0%)] in ITS−rDNA, 17/516 bp (3.29%) in *GAPDH*, and 8/744 bp (1.07%) in *TEF1*; and from *B. simmondsii* (BRIP 12030) by 4/500 bp [0.80%, with three gaps (0%)] in ITS−rDNA, 9/545 bp (1.65%) in *GAPDH*, and 3/744 bp (0.40%) in *TEF1*. The PHI analysis further confirms that *B. iranica* has no significant genetic recombination with closely related species (Φw = > 0.05, **Figure 11**). Morphologically, *Bipolaris persica* can be differentiated from closely related taxa by its longer conidiophores (up to 350 μm *vs.* up to 240 μm in *B. simmondsii*), and by its accentuated septa, which are absent in *B. heveae*, *B. microlaenae*, and *B. simmondsii* (Manamgoda et al., 2014; Tan et al., 2016). However, *B. persica* shares overlapping morphological characteristics and the same host with *B. iranica*, complicating their differentiation. Unlike *B. persica*, *B. iranica* has a broader host range, including *Arundo donax*, *Cynodon dactylon*, *Echinochloa colona*, *Hordeum vulgare*, *Sorghum halepense*, and *Triticum aestivum* (this study). Consequently, using molecular tools is essential for accurately distinguishing *Bipolaris* species and identifying any cryptic species.

**Figure 13:**
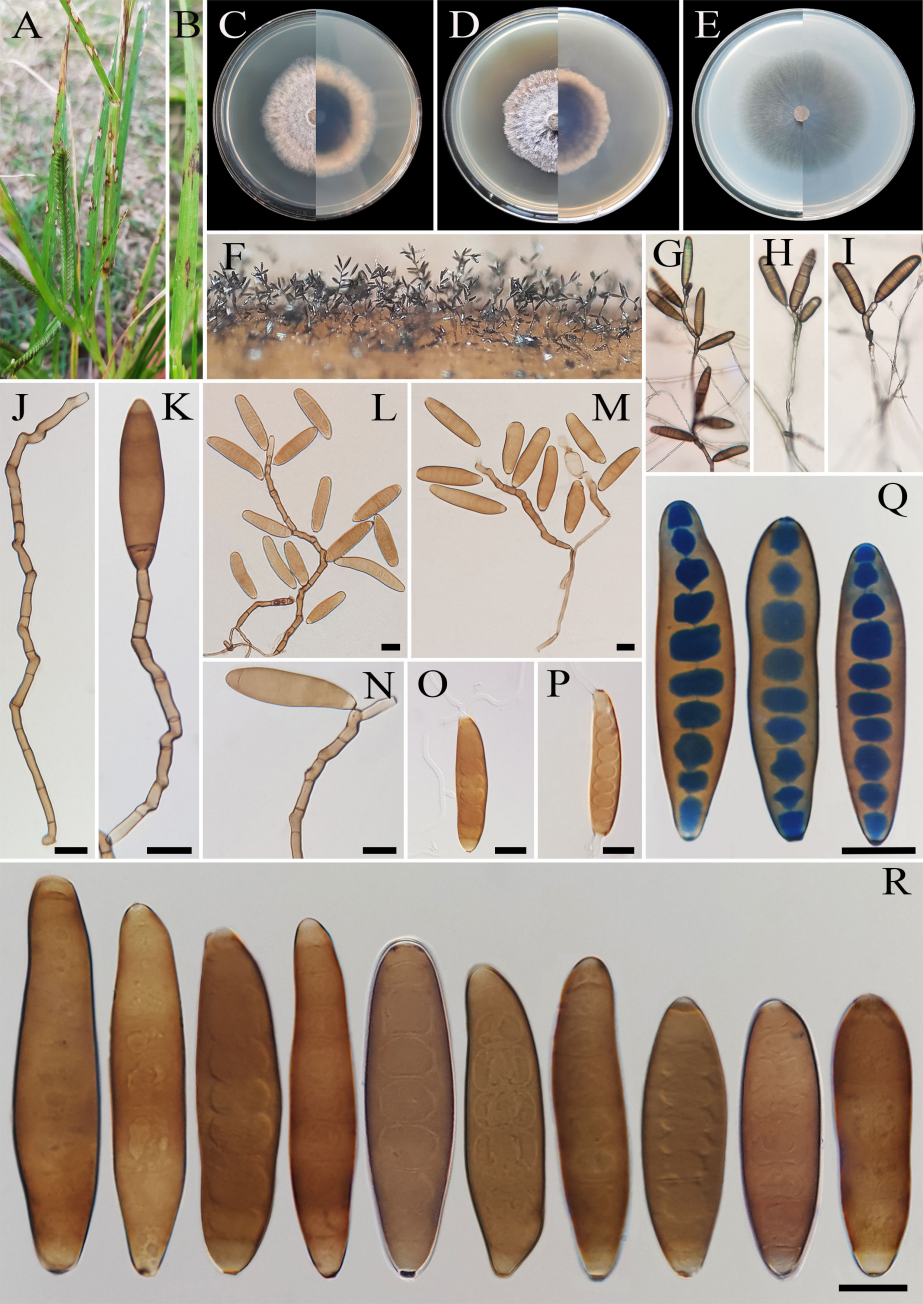
*Bipolaris crotonis* (IRAN 4807C). **(A, B)** Lesions on host leaf (*Eleusine indica*). **(C−E)** Colonies (front and reverse) on PDA **(C)**, MEA **(D)**, and CMA **(E)** media after 7 days. **(F−I)** Sporulation pattern on TWA medium. **(J–N)** Conidiophores. **(O, P)** Germinated conidia. **(Q, R)** Conidia. **(J−R)** Scale bars = 20 μm.

***Bipolaris crotonis*** Sivan., Trans. Br. mycol. Soc. 84(3): 404 (1985) (**Figure 13**).

*Description*: Leaf spots on *Eleusine indica*, gray at the center and dark brown margins. Sexual morph: Undetermined. Asexual morph: On TWA *Hyphae* 3- to 6-μm wide, pale brown to brown, smooth, septate, branched. *Conidiophores* (60–)100–300(–325) × 5–7 µm ( $\bar{x}$ ± SD = 200± 100 × 6 μm, *n* = 50), mononematous, semi- to macronematous, arising singly or mostly in groups, unbranched, straight to flexuous, septate, geniculate, pale brown to brown, paler toward the apex, swollen at the base. *Conidiogenous cells* (9–)11–26(–30) × 5–8 μm ( $\bar{x}$ ± SD = 18.5 ± 7.5 × 6.5 ± 1.5 μm, *n* = 50), mono- to polytretic, proliferating sympodially, integrated, terminal or intercalary, subcylindrical to slightly swollen, hyaline to pale brown, smooth walled to slightly verruculose, with thickened and darkened scars. *Conidia* (62–)75–100(–120) × 17–25 µm ( $\bar{x}$ ± SD = 87.5 ± 12.5 × 21 ± 4 μm, *n* = 50), straight, brown to dark golden brown, smooth walled, broadly ellipsoidal, fusoid to obclavate, tapering toward the rounded ends, apical and basal cells paler than the median cells, (5–)7–9(–11)-distoseptate, germinated mono- or bipolar; hila 2- to 3-μm wide, truncate, slightly protruding, thickened, and darkened. *Stroma*, *chlamydospores*, and *microconidiation* were not observed.

*Culture characteristics*: Colonies on PDA reaching 45 mm in diameter after 7 days at 25°C in the dark, circular, margin entire, velvety, gray at the center and white at the margin, with sparse gray aerial mycelia; reverse brown to pale brown from the center to the margin. Colonies on MEA reaching 40 mm in diameter, circular, margin entire, cottony appearance, gray at the center, white at the margin, with floccose aerial mycelia; reverse brown to pale brown from the center to the margin. Colonies on CMA reaching 50 mm in diameter, hairy appearance, olivaceous gray, with sparse gray aerial mycelia; reverse olivaceous gray at the center and a hyaline margin.

*Material examined*: IRAN, Mazandaran Province, Nour County, from leaf spots of *Eleusine indica* (Poaceae, Poales), 10 September 2022, Hashemlou E., living culture IRAN 4807C.

*Hosts*: *Croton* sp., *Eleusine indica* (Sivanesan, 1987; Manamgoda et al., 2014; Farr et al., 2024).

*Distribution*: Australia, Iran (this study), Papua New Guinea, Samoa, and Vanuatu (Sivanesan, 1987; Manamgoda et al., 2014; Farr et al., 2024).

*Notes: Bipolaris crotonis* is morphologically similar and phylogenetically related to *B. sorokiniana* (**Figure 1**) and can be differentiated by having longer conidia [(51–)60–110(–138) × (14–)20–25(–32) µm *vs.* (31–)40–72(–100) × 15–25(–27) μm in *B. sorokiniana*] (Sivanesan, 1987; Manamgoda et al., 2014). This species is heterothallic, and sexual morph can be developed by crossing compatible isolates in Sach^’^s agar medium (Manamgoda et al., 2014). Tan et al. (2014) reported that *B. eleusines* is phylogenetically similar to *B. crotonis* and synonymized it under *B. crotonis* based on nomenclatural priority. *Bipolaris crotonis* was first reported on decaying leaves of *Croton* sp. (Euphorbiaceae) (Sivanesan, 1985; Manamgoda et al., 2014) and was later identified on *Eleusine indica* (Poaceae) (Tan et al., 2014; Bhunjun et al., 2020). This species has also been reported as the causal agent of black point disease in wheat on the North China Plain (Xu et al., 2018) and as an endophytic fungus associated with *Dillenia indica*, an ethnomedicinal plant (Prasher and Kumar, 2021). To the best of our knowledge, this is the first report of *B. crotonis* in Iran.

**Figure 14:**
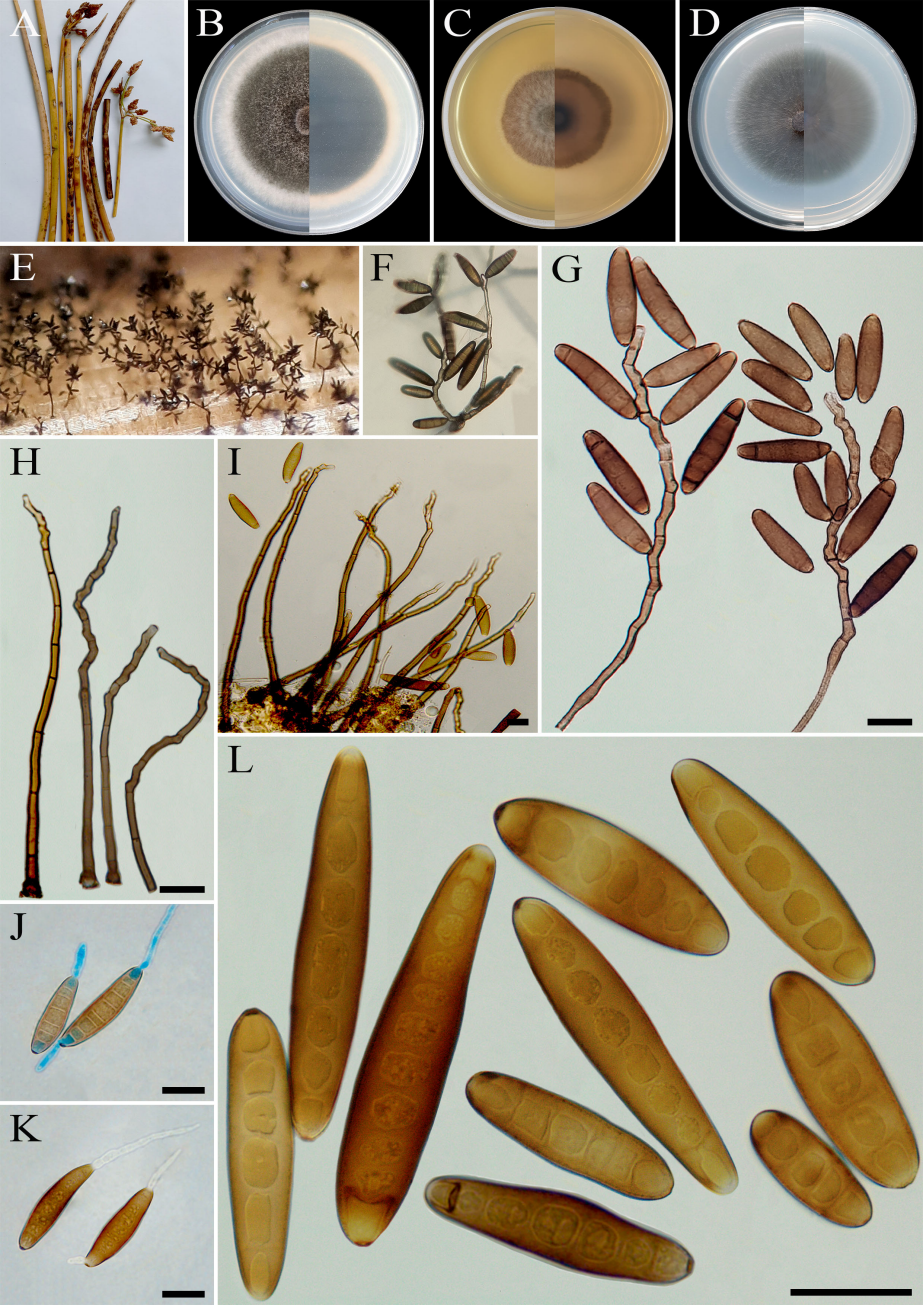
*Bipolaris salkadehensis* (IRAN 3382C). **(A)** Lesions on host culms (*Scirpus acutus*). **(B−D)** Colonies (front and reverse) on PDA **(B)**, MEA **(C)**, and CMA **(D)** media after 7 days. **(E, F)** Sporulation pattern on TWA medium. **(G–I)** Conidiophores. **(J, K)** Germinated conidia. **(L)** Conidia. **(G−L)** Scale bars = 20 μm.

***Bipolaris salkadehensis*** Ahmadpour & Heidarian, Mycotaxon 120: 302 (2012) (**Figure 14**).

*Description*: Culm spots on *Scirpus acutus*, 1- to 20-mm long, with dark brown spots. Sexual morph: Undetermined. Asexual morph: on TWA *Hyphae* 3- to 5-μm wide, pale brown to brown, smooth, septate, branched. *Conidiophores* (225–)260–400(–590) × 6–7 µm ( $\bar{x}$ ± SD = 330 ± 70 × 6.5 ± 0.5 μm, *n* = 50), mononematous, semi- to macronematous, arising singly or in groups, unbranched, straight to flexuous, septate, geniculate, pale brown to brown, paler toward the apex, swollen at the base. *Conidiogenous cells* (8–)10–24(–30) × 5–8 μm ( $\bar{x}$ ± SD = 16 ± 6 × 6.5 ± 1.5 μm, *n* = 50), smooth walled, mono- to polytretic, proliferating sympodially, integrated, terminal or intercalary, subcylindrical to slightly swollen, subhyaline or pale brown to brown. *Conidia* (32–)52–70(–93) × 11–15 µm ( $\bar{x}$ ± SD = 61 ± 9 × 13 ± 2 μm, *n* = 50), brown to dark brown, smooth walled to slightly verruculose, straight to slightly curved, subcylindrical to fusoid, occasionally obclavate to clavate, tapering toward the rounded apex, median cells brown to dark brown, apical and basal cells paler than the median cells being subhyaline to pale brown, end cells often cut off by a thick dark septum, (3–)5–8(–10)-distoseptate, germinated mono- or bipolar; hila 2- to 4-μm wide, flat to slightly protuberant, thickened, and darkened. *Stroma*, *chlamydospores*, and *microconidiation* were not observed.

*Culture characteristics*: Colonies on PDA reaching 60 mm in diameter after 7 days at 25°C in the dark, circular, margin entire, olivaceous gray to olivaceous brown with white to gray aerial mycelia; reverse gray olivaceous to olivaceous black at the center, white at the margin. Colonies grow more slowly on MEA, reaching 40 mm in diameter, circular, with irregular margin, gray at the center, white at the margin, with floccose aerial mycelia; reverse brown. Colonies on CMA reaching 51 mm in diameter, hairy appearance, olivaceous gray, with sparse gray aerial mycelia; reverse olivaceous gray.

*Materials examined*: IRAN, West Azarbaijan Province, Khoy County, on infected culms of *Scirpus acutus* (Cyperaceae, Poales), 10 September 2020, A. Ahmadpour, living culture IRAN 3382C; *ibid.* on infected leaves of *Sparganium erectum* (Typhaceae, Poales), 20 September 2010, A. Ahmadpour, living culture BI 1 = IRAN 3385C; *ibid.* on infected leaves of *Cladium mariscus* (Cyperaceae, Poales), 20 September 2010, A. Ahmadpour, living culture BI 4 = IRAN 3386C; *ibid.* on infected leaves of *Setaria* sp. (Poaceae, Poales), 25 September 2013, A. Ahmadpour, living culture FCCUU 1002; West Azarbaijan Province, Miyandoab County, on infected leaves of *Sorghum halepense* (Poaceae, Poales), 10 July 2013, A. Ahmadpour/Z. Heidarian, living culture IRAN 3383C; *ibid.* on infected leaves of *Arundo donax* (Poaceae, Poales), 10 July 2013, A. Ahmadpour/Z. Heidarian, living culture FCCUU 1001; *ibid.* on infected leaves of *Hordeum vulgare* (Poaceae, Poales), 10 May 2014, A. Ahmadpour/Z. Heidarian, living culture FCCUU 1003.

*Hosts*: *Arundo donax*, *Cladium mariscus*, *Hordeum vulgare*, *Scirpus acutus*, *Setaria* sp., *Sorghum halepense*, and *Sparganium erectum* (Ahmadpour et al., 2012a; Farr et al., 2024; this study).

*Distribution*: Iran (Ahmadpour et al., 2012a; this study).

*Notes: Bipolaris salkadehensis* was originally reported from infected leaves of *Sparganium erectum* (Sparganiaceae) and *Cladium mariscus* (Cyperaceae) with brown oval to elliptical lesions based on morphological characteristics and molecular data obtained from ITS−rDNA sequences (Ahmadpour et al., 2012a). In this study, two additional genes, *GAPDH* and *TEF1*, were sequenced for the ex-type isolate (Bi 1 = IRAN 3385C) as well as for other isolates from various plant hosts (**Table 1**) and used in phylogenetic analyses. *Bipolaris salkadehensis* isolates clustered well in a separate lineage with 100% ML, 100% MP bootstrap, and 1.0 BI posterior probability values (**Figure 1**). This species is morphologically similar to *B. cynodontis* and *B. setariae* (Sivanesan, 1987; Ahmadpour et al., 2012a). However, the conidia of *B. cynodontis* are smaller in size (30–75 × 10–16 μm), have three to nine (commonly seven to eight) distoseptate, and without cut-off in end cells (Sivanesan, 1987; Ahmadpour et al., 2012a). The conidia of *B. setariae* are fusoid to navicular, pale to mid golden brown, 5–10 distoseptate, 45–100 (mostly 50–70) × 10–15 μm, without cut-off in end cells, and lighter than those of *B. salkadehensis* (Sivanesan, 1987; Ahmadpour et al., 2012a). Zibani et al. (2025) have reported that *B. salkadehensis* exhibits low virulence as a pathogen of corn in Algeria. To the best of our knowledge, *Arundo donax*, *Hordeum vulgare*, *Scirpus acutus*, *Setaria* sp., and *Sorghum halepense* are newly identified hosts for this species.

## 4 Discussion

Advancements in molecular biology and phylogenetics have revolutionized our understanding of *Bipolaris* taxonomy and phylogeny. Techniques, such as DNA sequencing and phylogenetic analysis have enabled the identification of cryptic species and elucidated evolutionary relationships within the genus (Manamgoda et al., 2012, 2014; Tan et al., 2016; Raza et al., 2019; Bhunjun et al., 2020; Ferdinandez et al., 2022). Nevertheless, challenges persist in defining species boundaries and integrating morphological and molecular data. The results of this study revealed the presence of seven previously undocumented *Bipolaris* species from Iran, representing significant additions to the fungal biodiversity of the region. Comprehensive morphological characteristics and molecular phylogenetic analyses distinguish these new species from known taxa within the *Bipolaris* genus. Moreover, the study reports new records of *Bipolaris* species (*B. crotonis*) and identifies additional plant hosts for *B. salkadehensis*, highlighting the ecological diversity and host specificity of these fungi in Iranian ecosystems. Understanding their distribution, host range, and pathogenicity is crucial for developing effective disease management strategies and safeguarding agricultural crops in Iran.

Numerous *Bipolaris* species exhibit similar morphological characteristics making identification based solely on morphology unreliable and often ambiguous. Factors, such as environmental conditions, host plants, substrate, and culture media, further influence the morphological characteristics of *Bipolaris* species (Sivanesan, 1987; Alcorn, 1988; Manamgoda et al., 2012, 2014, 2015; Tan et al., 2014, 2016; Marin-Felix et al., 2017a, b, 2020). Accurate identification and understanding of genetic and pathogenic variability are essential for developing effective control measures and breeding programs. Molecular analyses, particularly using ITS−rDNA, *GAPDH*, and *TEF1* loci, have proven invaluable in addressing these challenges, offering more precise species delimitation within the *Bipolaris* genus (Berbee et al., 1999; Manamgoda et al., 2012, 2014, 2015; Tan et al., 2014, 2016; Marin-Felix et al., 2017a, b, 2020). While ITS−rDNA has limitations in distinguishing closely related species, *GAPDH* has been shown to be a more informative genetic marker and is recommended as a critical locus and supplementary barcode for accurately identifying closely related *Bipolaris* species (Manamgoda et al., 2012, 2014, 2015; Madrid et al., 2014; Tan et al., 2014, 2016; Marin-Felix et al., 2017a, b, 2020; Bhunjun et al., 2020). Comparative analyses of taxa have consistently demonstrated that the *GAPDH* gene region provides greater resolution, as indicated by multiple prior investigations (Berbee et al., 1999; Manamgoda et al., 2012, 2014, 2015; Madrid et al., 2014; Tan et al., 2014, 2016; Ferdinandez et al., 2022). In our study, *B. persica* and *B. iranica* exhibit overlapping morphological features complicating their differentiation. Therefore, molecular tools are essential for accurate differentiation among *Bipolaris* species.

The sexual morph of the fungus *Bipolaris* is rare in natural environments but has been observed under controlled laboratory conditions (Sivanesan, 1987; Manamgoda et al., 2011, 2014). The majority of *Bipolaris* species are heterothallic, with their sexual reproduction determined by mating-type idiomorphs, *MAT1-1* and *MAT1-2* (Turgeon, 1998; Yun et al., 1999; Lu et al., 2011). In heterothallic species, the presence of both mating types is necessary for the development of sexual structures. The only previously known homothallic species within the *Bipolaris* genus is *B. luttrellii* (synonym: *Cochliobolus luttrellii*), which contains both *MAT1-1* and *MAT1-2* idiomorphs (Turgeon, 1998; Yun et al., 1999; Lu et al., 2011). This study identifies *B. hedjaroudei* as another homothallic species capable of forming a sexual morph on TWA medium supplemented with host leaves (**Figure 8**) making it the second known homothallic species in the genus. *MAT* genes are particularly useful for studying the evolution of reproductive strategies and sexual mechanisms, as they appear to evolve faster than ITS−rDNA and *GAPDH* sequence regions (Turgeon, 1998). Additionally, due to their high interspecies variability and low intraspecies variability, *MAT* genes have been proposed as potential markers for defining species boundaries (Yun et al., 1999). Phylogenetic analyses of *MAT* genes, along with ITS−rDNA and *GAPDH* sequences, provide valuable insights into the evolutionary history of reproductive strategies (Turgeon, 1998). Yun et al. (1999) also developed a specific PCR assay for amplifying *MAT* idiomorphs using the TAIL–PCR technique. Further research should focus on identifying and characterizing the mating type idiomorphs in *B. hedjaroudei* and other newly identified species in this study.

Most of the newly identified *Bipolaris* species in this study, including *Bipolaris avrinica*, *B. azarbaijanica*, *B. banihashemii*, and *B. hedjaroudei*, were isolated from *Setaria* species. This plant genus is among the most significant weeds affecting field crops in Iran. Some species of *Bipolaris* (*B. bicolor*, *B. setariae*, and *B. sorokiniana* on *Eleusine indica*; *B. euphorbiae* on *Euphorbia heterophylla*; *B. eleusines* on *Echinochloa crus-galli*; *B. sorghicola* on *Sorghum halepense*; *B. yamadae* on various poaceous plants; and *B. microstegii*, *B. panici-miliacei*, and *B. zeicola* on *Microstegium vimineum*) are known to cause diseases in weeds. These fungi have shown potential for use as herbicides and have been validated as effective mycoherbicides in several studies (Figliola et al., 1988; Winder and Van Dyke, 1990; Kleczewski and Flory, 2010; Manamgoda et al., 2011; Zhang et al., 2014, 2022; Omar and Naqiuddin, 2020; Tan et al., 2022, 2024; Xiao et al., 2022; Khan et al., 2023). Additional research is needed to investigate the host range, pathogenicity, and potential applications of these newly discovered species as mycoherbicides on *Setaria* spp. and other plants within the Poaceae family. Recent studies suggest that certain phytopathogenic fungi can switch hosts and infect nearby plants (Rai and Agarkar, 2016). Weeds can serve as reservoirs for pathogens that threaten economically important crops. Additionally, environmental changes may drive certain fungi, previously considered mildly pathogenic, to evolve into more aggressive pathogens in new hosts (Manamgoda et al., 2011; Rai and Agarkar, 2016; Hernández-Restrepo et al., 2018). This study also revealed that *B. iranica* and *B. salkadehensis* have wide host ranges, being isolated from barley, wheat, and various weed species. Hence, the accurate identification of *Bipolaris* species associated with cereal crops and their weedy hosts is crucial for effective disease management and for ensuring stable crop production.

Understanding fungal biodiversity, such as *Bipolaris* species, is crucial for understanding diverse ecosystems globally. Exploring their diversity, distribution, and ecological functions enhances our understanding of plant health and ecosystem dynamics on a worldwide scale. This comprehensive study highlights the rich diversity of *Bipolaris* species and the complex relationships between fungi and their environments providing insights that are valuable for developing strategies to manage plant diseases and conserve fungal biodiversity in different regions.

## 5 Conclusions

This study analyzed 85 *Bipolaris* isolates collected from various hosts, in the Poales and Asparagales plant orders, across different locations in Iran between 2010 and 2022. Seven new species (*B. avrinica*, *B. azarbaijanica*, *B. banihashemii*, *B. hedjaroudei*, *B. hemerocallidis*, *B. iranica*, and *B. persica*) were discovered, along with two new records to Iran’s funga (*B. crotonis* and *B. salkadehensis*), identified through a combination of morphological characteristics and multi-locus phylogenetic analyses (ITS−rDNA, *GAPDH*, and *TEF1*). Investigating the diversity, distribution, and ecology of *Bipolaris* species in Iran is crucial for understanding their role in plant diseases, ecosystem interactions, and the development of effective disease management strategies in agriculture. This detailed study provides valuable insights into the diverse *Bipolaris* species in Iran paving the way for future research into plant diseases and fungal biodiversity in the country.

## Data Availability

The datasets presented in this study can be found in the online repository https://www.ncbi.nlm.nih.gov/genbank/ and the accession numbers are mentioned in **Table 1**.
